# The Curious Case of the HepG2 Cell Line: 40 Years of Expertise

**DOI:** 10.3390/ijms222313135

**Published:** 2021-12-04

**Authors:** Viktoriia A. Arzumanian, Olga I. Kiseleva, Ekaterina V. Poverennaya

**Affiliations:** Institute of Biomedical Chemistry, 119121 Moscow, Russia; olly.kiseleva@gmail.com (O.I.K.); k.poverennaya@gmail.com (E.V.P.)

**Keywords:** HepG2 cell line, mutations, hepatocellular carcinoma, hepatoblastoma, hepatocytes

## Abstract

Liver cancer is the third leading cause of cancer death worldwide. Representing such a dramatic impact on our lives, liver cancer is a significant public health concern. Sustainable and reliable methods for preventing and treating liver cancer require fundamental research on its molecular mechanisms. Cell lines are treated as in vitro equivalents of tumor tissues, making them a must-have for basic research on the nature of cancer. According to recent discoveries, certified cell lines retain most genetic properties of the original tumor and mimic its microenvironment. On the other hand, modern technologies allowing the deepest level of detail in omics landscapes have shown significant differences even between samples of the same cell line due to cross- and mycoplasma infection. This and other observations suggest that, in some cases, cell cultures are not suitable as cancer models, with limited predictive value for the effectiveness of new treatments. HepG2 is a popular hepatic cell line. It is used in a wide range of studies, from the oncogenesis to the cytotoxicity of substances on the liver. In this regard, we set out to collect up-to-date information on the HepG2 cell line to assess whether the level of heterogeneity of the cell line allows in vitro biomedical studies as a model with guaranteed production and quality.

## 1. Introduction

Cell lines have revolutionized scientific research because of their similarity to primary tissues, low cost, and ease of use and culture. In addition, such cells provide an unlimited supply of biomaterials, and their use in research avoids ethical problems associated with the utilization of animal and human tissues [1]. The widespread use of cell lines has been found in the production of vaccines [2], cytotoxicity testing [3,4] and the identification of drug metabolic pathways [5,6], the study of gene function [7], the creation of artificial tissues (for example, artificial skin [8]), and the synthesis of therapeutic proteins [9,10].

The liver is an organ with high regenerative capacity and complex functions [11]. It maintains various physiological processes [12], predominantly replenishing and storing rapidly mobilized energy reserves in glycogen, regulating carbohydrate metabolism, and neutralizing and removing metabolic products from the body [13]. In addition, the liver takes part in the metabolism of nutrients, receiving digestive products in the form of glucose, amino acids, fatty acids, and glycerol. Moreover, there are many different types of liver diseases. These can be inherited (Wilson disease, hemochromatosis, and alpha 1-antitrypsin deficiency) [14] or acquired (such as hepatitis and liver cancer) [15,16].

In many studies, immortalized hepatic cell lines are used instead of liver biopsies due to their high cost, high invasiveness, and reduced activity of several key enzymes. Moreover, there is no technique enabling the maintenance of liver biopsies in culture for time-consuming studies [17]. At the same time, based on the descriptions in the databases, the hepatic cell lines are described as “epithelial morphology”. Thus, the human cell lines THLE-2 and THLE-3, considered models of normal hepatocytes, are controversial. Epithelial cells include hepatocytes and cholangiocytes. These cells perform different functions, with hepatocytes responsible for the formation of bile; the metabolism of glucose, amino acids and lipids; the detoxification of bilirubin and ammonia; and the production of serum proteins [3,18]. Cholangiocytes, in turn, modulate the composition of bile, secrete chloride and carbonate ions, and secrete and absorb water when bile passes through the intrahepatic bile duct [19]; in the event of a malfunction of hepatocytes, cholangiocytes replace them [20,21]. However, the article describing the characteristics of the THLE-2 and THLE-3 cell lines mentioned their similarity with human hepatocytes [22]. Such inaccuracies in wording can affect the design of studies and the interpretation of experiments designed and performed to answer the biomedical question.

**Figure 1:**
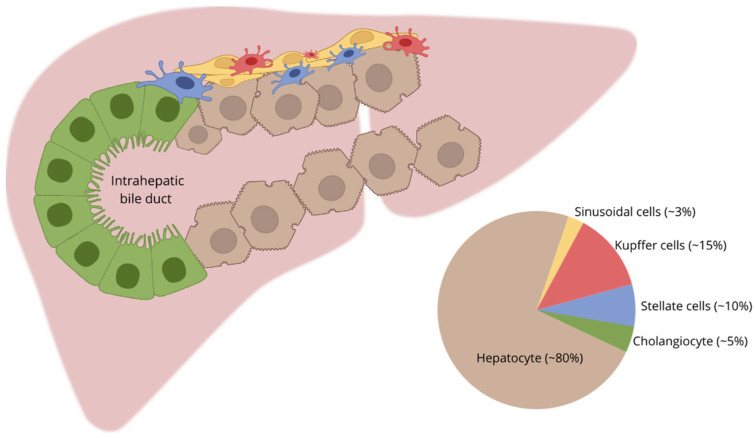
Types of human liver cells and their distribution.

It should also be noted that, in addition to epithelial cells, the liver includes Kupffer cells (~15% of the total liver cell population), stellate cells (~8–10%), and liver sinusoidal endothelial cells (~3%) (see Figure 1). This diversity presents researchers with the daunting task of creating a liver model, both in vivo and in vitro, that would include all types of cells [23].

The use of immortalized hepatic tumor cell lines has become a widespread practice not only for the cancer research, but also for the study of hepatitis B (HBV) and hepatitis D (HDV) viral infections. Thus, the complete cell cycle of HDV replication and expression/replication of HBV were found in cell lines HepG2 and Huh7 [24,25]. Additionally, mechanisms of HBV viral entry were discovered in HepaRG cell lines [26].

**Figure 2:**
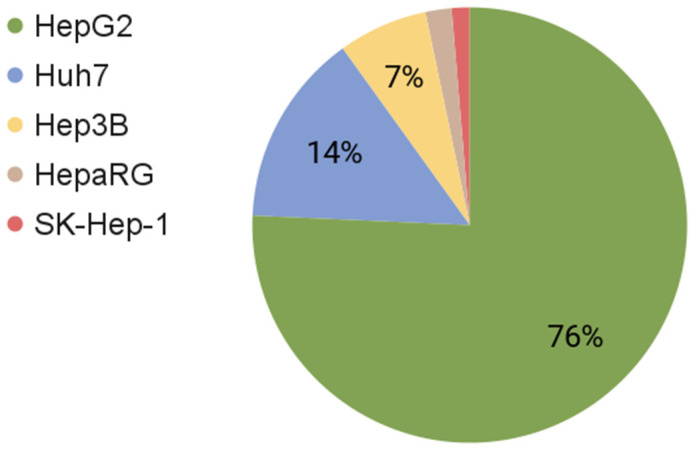
Percentage of manuscripts using human hepatic cell lines in accordance with the corresponding search term on PubMed (accessed on 23 November 2021).

Currently, there are about 40 various hepatic tumor cell lines, but the most commonly used are HepaRG, Huh7, SK-Hep-1, Hep3B, and HepG2, obtained from various tumors [20] (Figure 1). Among the cell culture mentioned above, the HepG2 cell line has gained popularity due to its wide range of applications in scientific research. Therefore, at the time of writing, the database PubMed (https://pubmed.ncbi.nlm.nih.gov/, accessed on 23 November 2021) contained a total of 34,021 articles available after a search for “HepG2”; 6455 for the Huh7 cell line; 2994 articles for the Hep3B cell line; 876 about HepaRG; and 625 articles on SK-Hep-1 (Figure 2).

A meta-analysis of the articles showed that each of the cell types comes from a specific type of cancer. For example, the Huh7 cell line has the characteristics of HCC [27], MT-CHC01 originates from ICCA cells [28], and HepG2—HB [29]. In Table 1, we summarize the characteristics of the top five most studied hepatic tumor cell lines. Characteristics for all hepatic cell lines are presented in Supplementary Material Table S1.

This review provides generalized information on the HepG2 cell line, examples of its use, assessment of its characteristics, and its applicability as model objects.

## 2. Historical Background

Among hepatic cell lines, HepG2 cells were the first to exhibit the key characteristics of hepatocytes. This line was isolated in 1975 and described as hepatocellular carcinoma (or hepatoma, HCC) [41]. On the other hand, the earlier cell line SK-Hep1, created in 1971, although considered a model of HCC, does not possess critical markers of liver cells, including the expression of albumin and alpha- and gamma-fibrinogen [42].

A patent for the HepG2 cell line, “a human hepatoma-derived cell line”, was filed in 1980 by researchers at the Wistar Institute. Since then, HepG2 cells have been entered into the ATCC (American Type Culture Collection, Rockville, MD, USA) repository as a human cell line (HB 8065), “derived from the liver tissue of a 15-year-old white male with a well-differentiated hepatocellular carcinoma” [29].

As in several cases, a classification error crept in, and the HepG2 cell line was mistakenly labeled as hepatocellular carcinoma (HCC) instead of hepatoblastoma (HB), which caused considerable confusion for 30 years. This was discovered after an investigation by Lopez-Terrada D. et al. [29] on the nature of HepG2. In addition to visual cytological signs of the original cell preparations, from the molecular side, losses in the chromosome 4q3 region were found, which are associated with t(1; 4) translocation—a common occurrence with HB [43], as well as with other characteristic HB chromosomal abnormalities, including trisomies 2 and 20 [29]. Other signs of an HB nature include the corresponding parameters of the Wnt/β-catenin signaling pathway and dysregulation of cell growth and survival pathways such as fetal and embryonal HB [44], a characteristic deletion of the third exon of the CTNNB1 gene, which is identical to that described in epithelial type HB, as well as similar gene expression profiles in HepG2 and tumor cells in hepatoblastoma [29].

Regarding the differences between the HepG2 cell line and normal hepatocytes, the crucial point is the weak or absent expression of the cytochrome P450 (CYP) superfamily, CYP3A4, CYP2C9, CYP2C19, CYP2A6, CYP2D6, etc. [45], which are involved in phase 1 xenobiotic oxidation in the liver. Nevertheless, it is believed that the HepG2 cell line has retained most of the metabolic functions of normal hepatocytes; therefore, it is used to study the toxic effects of heavy metals, nanoparticles, and drugs in vitro [46].

## 3. Comparison of Liver Cancer

One of the possible reasons for the popularity of the use of the HepG2 cell line is related to the fact that most cases of primary liver cancer are hepatocellular carcinoma [47]. Liver cancer is the third leading cause of cancer death worldwide [48]. There are several types of primary liver cancer: HCC (80–90% of cases), intrahepatic cholangiocarcinoma (ICCA, 10–15% of cases), HB and angiosarcoma (AS) [49]. It should be noted that hepatoblastoma is the most common malignant liver tumor in children; it accounts for about 70% of cases, followed by HCC, accounting for 27% [50].

**Figure 3:**
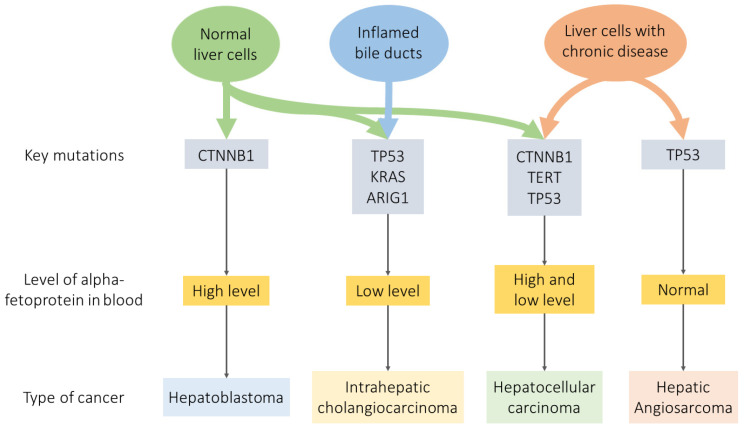
Significant liver cancer markers.

Each type of liver cancer and its subtype differs in cytological and molecular characteristics (Figure 3). As mentioned above, one of the hallmarks of HB at the molecular level is damage to the CTNNB1 gene encoding β-catenin in the third exon in the form of deletions or insertions [51], but most often, the loss of serine/threonine residues (in codons S33, S37, S45 and T41), as well as the substitution of tyrosine for alanine (T41A) [52]. The frequency of mutations of this gene is observed in 50–90% of patients [51,53]. It should be noted that in the case of HCC, mutations in CTNNB1 can also be observed, but they are much less common (20–40% of patients [54]) and are represented by changes in codons 32, 33, 38, and 45 [55]. Mutations in β-catenin lead to its accumulation in tumor cells due to the loss of original function, i.e., impairment of the Wnt/β-catenin signaling pathway, which plays a vital role in the development, regeneration, and metabolic zoning process of the liver [56].

The key features of HCC [57,58] and several oncological diseases [59] are a mutation of the TERT gene promoter, which triggers the activation of telomerase reverse transcriptase, the process of tumor formation. Promoter mutations have been identified as the most frequent genetic changes in HCC, with an overall incidence of about 30–60% [60]. The second most frequent disorder in HCC is mutations in the tumor suppressor gene TP53 [61], some mutations of which contribute to the emergence of the pro-oncogenic function of the encoded protein p53—increased cell proliferation, drug resistance, and increased migration and invasion of cells, as well as the stimulation of neoangiogenesis [62,63]. Such hot-spot mutations (in particular, R249S and V157F) are associated with a poor prognosis for patients with HCC [64].

Hepatic AS and ICCA also have a TP53 gene mutation associated with reduced survival [65,66,67]. In ICCA, mutations are also often observed in the KRAS and ARID1A genes [68,69], the proteins involved in the cell signaling pathways that control cell growth, maturation, and death. Sometimes, mutations occur in genes involved in Wnt signaling, i.e., CTNNB1, AXIN1, and APC [70,71].

One of the biomarkers of tumor processes in the liver is a change in the level of alpha-fetoprotein (AFP)—plasma protein produced by the yolk sac and the fetal liver during fetal development [72,73]. An increase in the AFP level promotes the proliferation of tumor cells and the formation of blood vessels and enhances the antiapoptotic effect of cancer cells [74]. Changes in the AFP level also predict the course of disease, the therapeutic response, and the occurrence of relapse [75].

Thus, existing differences and similarities in the molecular characteristics of liver cancer types raise a logical question about the applicability of the HepG2 cell line as a model of normal and pathological cellular processes in each case.

## 4. Comparison of HepG2, Normal Hepatocyte, HB, and HCC

According to their cytological characteristics, HepG2 cells are certainly the most similar to tumor cells in hepatoblastoma, but at the same time, they retain features characteristic of normal hepatocytes (see Table 2). Thus, the average diameter of a HepG2 cell is about 10–20 µm, of a hepatocyte, 15 µm, and tumor cells with HB, 10–20 µm. We did not find exact data for HCC cells, but all tumor cells have a diameter >10 µm.

About 20% of human hepatocytes are binucleated or polyploid [76,77]; often, their nuclei are anisokaryotic. HepG2 cells contain three to seven nuclei [78], which account for up to 25% of the total cellular protein, although their size is somewhat more prominent than in normal hepatocytes, containing up to 10% of the total protein in the cell. In tumor cells, which are also characterized by an abnormal number of chromosomes, an increase in the number of nuclei is observed—up to seven per cell.

Hepatocytes are rich in the smooth endoplasmic reticulum (SER) and mitochondria, reflecting their intense protein synthesis and energy metabolism, respectively [79]. HepG2 SER cells are poorly developed, and the number of mitochondria is half that of hepatocytes. Ultrastructural analysis of cells in HB revealed well-formed intercellular junctions, numerous intermediate filaments, and rare cytoplasmic organelles: mitochondria and a rough endoplasmic reticulum [80]. In tumor cells with HCC, the numbers of mitochondria and endoplasmic reticulum are also significantly reduced, and they have an abnormal structure characteristic of stressful conditions.

Thus, according to the cytological characteristics, it can be noted that HepG2 cells occupy an intermediate state between normal hepatocytes and tumor cells, for which significant changes in the epigenetic regulation of nuclear and mitochondrial genes are observed [81,82].

### 4.1. Genome

Numerous studies have shown that this cell line contains fairly stable chromosomal abnormalities [30,83,84,85,86]. HepG2 cells contain translocations between the short arms of chromosomes 1 and 21 [86], trisomies of chromosomes 2, 16, and 17, and tetrasomy of chromosome 20 [30,87]. The number of chromosomes varies from 50 to 60 [13,14], corresponding to the hyperdiploid karyotype [88]. More than 100 of the chromosomes are observed in some cases, characterized by tetraploid enlargement [86,89]. The HepG2 cell contains about 7.5 pg of DNA, 15% more than in a normal somatic cell [12,90].

In addition to the marker mutations discussed above (see Figure 3) [30], in an article by Zhou et al. in which the genome of the HepG2 cell line was studied, 377 SNVs and 255 indels, which are private protein-altering (PPA), were found using whole-genome sequencing [30]. Some are accounted for among the characteristic mutations by well-known oncogenes and tumor suppressors, such as NRAS, STK11/LKB1, and PREX2, and several genes associated with tumor processes, CDK12 IKBKB and RP1L1.

In an article by Tianyou et al. that studied polymorphisms in the NRAS and KRAS genes in Chinese children, no significant association was found between the risk of developing hepatoblastoma and these polymorphisms [91]. There are also no data on the relationship of mutations in the PREX2 gene with hepatoblastoma; however, it has been shown that in 23.5% of patients with HCC, there is a non-silent somatic mutation of PREX2 [92]. A similar situation for the RP1L1 gene is defined as a driver in HCC [15,93,94,95], but there are no data on the relationship with the development of HB.

The HepG2 cell line carries a mutation in the TERT promoter that is common in HCC—C228T [96]. In HB, a somatic mutation is also observed in the promoter of this gene—C250T [51,52,97]. It should be noted that a mutation in the TERT promoter contributes to immortalization, protecting telomeres in cancer cells.

The TP53 gene can be found in liver cancer in two types: mutant and wild. Wild-type TP53 [98] is observed in the HepG2 cell line, as in HCC and HB [99]. In HCC, mutant TP53 tumors have higher malignant potentials than those with wild-type TP53 [100]. The TP53 gene is critical in suppressing cancer in humans, as it plays a role in cell cycle arrest, apoptosis, and ageing. Thus, this mutation in the gene can promote cell proliferation.

Thus, it is worth noting that parts of the driver mutations in the genes of the HepG2 cell line coincide with mutations in HCC and HB. Furthermore, a marker deletion in the reading frame of the CTNNB1 gene for HB also occurs in HCC in 19% of patients [54].

### 4.2. Transcriptome

A transcript is the next stage after the genome in the transfer of biological information, at which new characteristics appear, such as changes in the number of transcripts encoded by one gene and their expression. In total, about 14,000 genes are expressed in HepG2 cells [101]. At the transcriptome level for the HepG2 cell line, it has been shown that 50 genes are upregulated in comparison with normal hepatocytes [102]. Thus, genes associated with cancer have an increased expression level in the HepG2 cell line, and genes active in hepatocytes are associated with xenobiotic metabolism.

An analysis comparing results of the transcriptome profiling of hepatic cell lines and tumor cells in HB and HCC showed significant differences in the level of DLK1 gene expression in HepG2 cells [102,103,104], which may be necessary for driving chemoresistance and potentiating malignancy in cancers. A change in the level of DLK1 expression was also observed in tumor cells, and the gene was more actively expressed in HB cells than in HCC [44,105]. A similar pattern was found in the case of insulin-like growth factor 2 (IGF2) [106,107,108,109], which is associated with the development of various diseases in children—for example, Beckwith–Wiedemann syndrome [110,111,112,113]. Moreover, increased IGF2 expression is associated with a poor prognosis of the course of cancer because it supports both the proliferation and migration of tumor cells [114,115,116].

In HepG2, there is increased expression of the Wnt signaling pathway antagonist DKK1 [102], increasing the proliferation, colony-forming ability, and invasion. However, it is believed that altered DKK1 expression has no effect on tumor development in HB due to the accumulation of β-catenin [44]. In the case of HCC, overexpression of DKK1 leads to an increase in cell invasiveness through the MMP-7 signaling pathway, which is associated with poor prognosis [117].

Another example of a gene with altered expression is the GPC3 gene encoding glypican-3, which is overexpressed in HepG2 cells and tumor cells in HCC [118,119]. GPC3 is considered a potential biomarker for the early diagnosis of HCC [119,120]. When studying the functional response to the suppression of the GPC3 gene in HepG2 and Huh7 cells, a more pronounced response was revealed in the first case which, in both cases, consisted of a decrease in cell profiling [121].

In a study by Tyakht et al. [102], increased expression of genes encoding the insulin-like growth factor 2 mRNA-binding proteins, IGF2BP1 and IGF2BP3, was shown in HepG2 cells compared with normal hepatocytes. These genes are also overexpressed in HB [122,123], but in the case of HCC, a change in expression has only been shown for IGF2BP1, an increased value of which is associated with a poor prognosis for the course of the disease [124,125,126].

Compared with normal hepatocytes, HepG2 cells exhibit a decreased expression level of CYP genes [102,127,128], which play a decisive role in the metabolism of endogenous and exogenous molecules [128,129,130]. Moreover, reduced expression levels of these genes are also observed in tumor cells in HCC and HB, which, as expected, correlate with a low survival estimates [131].

Based on the analysis of transcriptome profiling data, common gene expression patterns are observed in HB, HCC, and HepG2 cells. The HepG2 has also been compared with other commonly used hepatic cell lines at the transcriptomic level. Thus, a study comparing HepG2 and HepaRG showed that the HB cell line has a greater affinity with normal hepatocytes [132]. However, the article by Jennen et al. noticed that HepaRG is more suited in an in vitro liver model for biological interpretations of the effects of exposure to chemicals, whereas HepG2—for classification studies using the toxicogenomics approach [133]. With a small proportion of genes differing in expression between HepG2 cells and normal hepatocytes, it is necessary to note significant functional differences in several genes responsible for metabolism and proliferation.

### 4.3. Proteome

The HepG2 cell contains, on average, 170 pg of total protein, which is almost three times less than in hepatocytes (600 pg) [12]. The liver is the main source of blood plasma proteins—fibrinogen, albumin, and globulins. Comparative analysis of the proteomic profile of hepatocytes and the HepG2 cell line showed that the titers of serum albumin (ALB), transferrin (AAA), and serpin (SERPINA1) did not differ. At the same time, plasma proteins such as ceruloplasmin (CP) and hemopexin (HPX) were detected in trace amounts or were not found at all in HepG2 cells [12,134].

According to the results of transcriptome profiling in HepG2 cells, the cytochrome P450 superfamily genes are weakly expressed, as expected—at the proteomic level, the corresponding proteins are either absent or found at very low concentrations [12]. Thus, it has been shown that CYP3A4, which is one of the key enzymes of drug cleavage [135], is 100–400 times less in HepG2 cells than in hepatocytes [12,136]. Low expression of these proteins was also observed in patients with HCC and HB [137,138].

A significant difference in the proteomic profiles of hepatocyte and HepG2 cells is also observed in the expression of phase II drug-metabolizing enzymes. Thus, the expression of enzymes of the UGT family—UGT1A1, UGT1A4, UGT1A6, UGT2B7, UGT2B15, and GSTM1, which play a key role in the metabolism of a wide range of anticancer agents—was observed either at very low levels in the HepG2 cell line or was not detected at all in comparison with hepatocytes [12,139]. It has been shown that UGT proteins are also weakly expressed in tumor cells during HCC [140].

For other phase II drug-metabolizing enzymes, SULT1A1 and SULT2A1, responsible for transferring a sulfate group from 3’-phosphoadenylyl sulfate to the hydroxyl group of an acceptor, no differences in titers were found between hepatocyte cells and HepG2. At the same time, it should be noted that in 50% of HCC cases, there is a decrease in the level of SULT2A1 [141,142]. Conjugates from phase II metabolism are excreted into the bile and/or the blood by efflux transporters in the canalicular and basolateral membrane of the hepatocytes, respectively [12]. This process is sometimes referred to as phase III metabolism. Most of the tubular outflow transporters are present in HepG2 cells at levels close to those found in hepatocytes. However, it has been shown that the titers of the BSEP protein, which carry bile salts, are 100 times lower in HepG2 cells than in hepatocytes [12,143], which is consistent with studies of the mRNA level [144]. In patients with HCC, the BSEP level is also decreased, which is associated with a poor prognosis of the course of the disease [145,146]. Of the basolateral efflux transporters MRP3, MRP4, and MRP6, MRP4 was not detected in HepG2 cells [12,147], and MRP3 and MRP6 titers were 4–20 times lower in HepG2 cells than in hepatocytes.

Thus, at the proteomic level, HepG2 cells retain and multiply changes in systems associated with the metabolism of endogenous and exogenous substances compared with normal hepatocytes. At the same time, the observed changes are similar to processes in hepatoblastoma and hepatocellular carcinoma cells, which justifies the use of the HepG2 cell line to study the metabolism of anticancer drugs and tumor processes.

### 4.4. Metabolome

Most of the metabolomic studies performed on the HepG2 cell line aim to investigate the effect of drugs and chemicals on cells. In total, a small part of the work was carried out on the comparison of HepG2 and other cell lines. In particular, Chen et al. (2018) [148] compared the hepatocyte cell lines L-02 and HepG2. Their study showed that in the HepG2 cell line, acetate, creatine, isoleucine, leucine, and phenylalanine were increased, which indicates a more enhanced lipid metabolism and active absorption of nutrients from the media than in L-02 cells. However, it should be noted that, according to the STR analysis, L-02 is a HeLa derivative and not a hepatic cell line [149], which casts doubt on the obtained assessment of the similarity of the HepG2 cell line with hepatocytes.

When comparing HepG2 cells with other tumor cell lines—MCF7 (angiosarcoma of the breast), PC3 (prostatic adenocarcinoma), 143B (osteosarcoma), and HEK293 (embryonic kidney)—it was shown that HepG2 cells had a decreased content of amino acids such as glutamine, proline, asparagine, aspartate, arginine, methionine, alanine, lysine, threonine, and leucine [150]. Moreover, HepG2 cells also had a high content of glutamate, phenylalanine glycine, acetylcarnitine, and methionine and choline derivatives [151]. Changes in amino acid levels affect protein synthesis, particularly the secretion of total hepatic protein, which shows a positive relationship between the concentration of several amino acids and the amount of total protein [152]. A low level of taurine was also observed in HepG2 cells, of which the primary role in the liver is conjugation with bile acids for excretion into bile [153]. The total amount of fatty acids was increased compared with the cell lines MCF7, PC3, 143B, and HEK293, and the hydrolysis of phospholipids was low, as assessed by glycerophosphoethanolamine, which may indicate the causes of degradation of the nascent very-low-density lipoproteins [154]. Thus, the observed metabolomic profile of HepG2 is generally characteristic of immortalized cells, but several differences are specific for the biological processes of hepatocytes.

The meta-analysis of the literature revealed almost no metabolic studies, including those comparing HCC and HB with hepatocytes and hepatic cell lines. Thus, due to the lack of relevant studies, we cannot conclude the similarity of HepG2 with other cells for use as models; however, the opportunity for further research is open.

### 4.5. Signalome

In the study of tumor processes, special attention is paid to signaling pathways that are responsible for the aberrant cell growth, survival, genome maintenance, etc. [155]. Signalome study provides another level for deciphering pathological processes and uses it to inform new treatments for precision medicine in cancer [156]. However, at the moment many aspects are still unknown, and the current level of information is limited.

The case of liver cancer focuses on the following pathways: the transforming growth factor β (TGF-β) [157], proto-oncogene Wnt/β-catenin [158], phosphoinositide 3-kinase (PI3K)/protein kinase B (Akt) [159], c-Jun N-terminal kinase (JNK)/signal transducer and activator of transcription (STAT) [160], Hedgehog and tumor protein 53 transduction pathways [161].

As noted above, one of the key features of HB due to the exon 3 mutation CTNNB1 gene is a violation of the Wnt/β-catenin signaling pathway [162]. This pathway is crucial in controlling hepatic homeostasis and in maintaining adherens junctions and metabolic zonation and regeneration, suggesting its role in almost every aspect related to liver functioning [162]. The same mutation is characteristic for the HepG2 cell line [29], with suppression of the Wnt/β-catenin signaling pathway expectedly leading to apoptosis [163]. Other altered pathways in HB are signal transducer and activator of transcription 3 (STAT3) [164] and signaling pathways PI3K/Akt, ERK, and p38 [165].

In the case of HCC, the major pathways involved in the oncogenic process, in addition, to Wnt/b-catenin [54], are Hedgehog, hepatocyte growth factor/c-MET, vascular endothelial growth factor (VEGF), mitogen-activated protein kinase (MAPK)/ERK (or Ras-Raf-MEK-ERK), and PI3K/AKT/Mtor [166]. Changes in the HepG2 cell line are observed in the transforming growth factor-beta (TGF-β) signaling pathway, which accounts for 38% of gene mutations in HCC [167]. Dysregulation of signaling in the TGF-β pathway plays a central role in inflammation, fibrogenesis, and immunomodulation in the HCC microenvironment [168].

### 4.6. HepG2 for Biomedical Research

Based on the meta-analysis of articles, we have summarized the recommendations for the use of the HepG2 cell line (Table 3). The table is based only on genomic and transcriptomic data since nucleic acid sequencing is more sensitive than mass spectrometry of proteins and metabolites [169]. Available experiments do not allow the whole picture of the proteome and metabolome to be seen [170,171].

## 5. Conclusions

Despite the illustrated differences between the HepG2 cell line and normal hepatocytes, the toxic effects of heavy metals, nanoparticles, and drugs are frequently studied in vitro [46,184,185,186,187].

The validity of using HepG2 cells as a model of hepatocytes is controversial because crucial proteins involved in the metabolism of substances—the liver’s primary function—are poorly expressed. Furthermore, the shortage of uptake transporters and phase I enzymes is observed in HepG2 cells, which indicates the need for careful use of this cell line to predict the metabolism and elimination of xenobiotics in hepatocytes. At the same time, the use of HepG2 cells to study the metabolism of anticancer drugs is acceptable because there is a similarity in the expression of phase I, II, and III drug metabolism/transport proteins in cells with HCC and HB. In addition, due to the low basal activity of CYP proteins (CYP1A2, CYP2B6, and CYP3A4), the cell line can be used in studies of CYP inducers [176].

Work is under way to modify the HepG2 cell line to increase the expression of cytochromes for the correct use of HepG2 cells as a model of hepatocytes in the study of drug metabolism [188]. Another approach is to derive three-dimensional spheroid cell cultures [189,190,191]. The three-dimensional culture method transforms cells into spheroids and creates a more physiologically relevant system. As a result, metabolic activity, including cytochromes, is higher in 3D spheroidal HepG2 models than in 2D cells [142], bringing it closer to normal hepatocytes.

According to molecular profiling data, the HepG2 cell line can serve as a model for hepatoblastoma. However, the use of HepG2 cells as an HCC model is incorrect, and as shown in the study by Choi et al. (2015), there is no expression of the marker of HCC—hGSTP1 [130].

For 40 years, the HepG2 cell line has been widely used as a model for normal hepatocytes, hepatocellular carcinoma, and hepatoblastoma cells in various studies. Researchers are studying its molecular composition at the genomic, transcriptomic, and proteomic levels, each time confirming that the HepG2 is only partially similar to primary hepatocytes and cancer cells. However, even after the publication of such articles, researchers continue to call the genesis of the cell line differently. The “surprising case” of the HepG2 cell line lies in its erroneous annotation. This is not critical for some work, such as studying the amount of protein in a cell. However, if the metabolism of drugs or the processes that occur in a particular type of cancer are being studied, this is important and may call into question the relevance of the study.

In this manuscript, we wanted to combine the accumulated knowledge about the cell line and compare it at different molecular levels with normal hepatocytes, HCC and HB. Hopefully, our review will simplify the scientists’ question: “Should I use HepG2 in my research?”

## Figures and Tables

**Table 1:** Characteristics of the top five most studied tumor hepatic cell lines.

	Cell Type	Mutated Genes	Number of Chromosomes	Number of Articles (PubMed)	Reference
HepG2	HB	CTNNB1	50–60	34,021	[12,30]
HepaRG	HCC	PLIN2, ANXA1, H2AFY, SNX1, GCHFR, APO	46	880	[26,31,32,33]
Huh7	HCC	KDR, POLD3, TERT, TP53	55–63	6463	[34,35,36,37]
Hep3B	HCC	AXIN1, RB1	≈60	2994	[34,38]
SK-Hep-1	Adenocarcinoma	CDKN2A, BRAF	56–64	602	[39,40]

**Table 2:** Comparison of HepG2 cells with hepatocytes and cells with HCC and HB.

	HepG2 Cells	Hepatocyte Cells	Cells with HB	Cells with HCC
Cell size and shape	12–19 µm, polygonal	15 µm, cube	10–20 µm, round or angulated	>10 µm, spindle-shaped and show bizarre anaplastic figures
Subcellular components	Large nuclei, 3–7 nucleoli, low mitochondrial content, and poorly developed SER ^1^	Two or more nuclei occupy 5–7% of the cell volume; high SER^1^ and mitochondria	Small, round, inconspicuous nucleoli; low mitochondrial and RER ^2^ content	The numbers of mitochondria and ER ^3^ is reduced, and have an abnormal structure, characteristic of stressful conditions
Number of chromosomes	50–60	Polyploidy	Aneuploidy, >46	Aneuploidy, >46
Genome stability, DNA content	7.5 pg genomic DNA, genome unstable	~6 pg genomic DNA, stable genome	Genome unstable	Genome unstable

^1^ SER, smooth endoplasmic reticulum; ^2^ RER, rough endoplasmic reticulum; ^3^ ER, endoplasmic reticulum.

**Table 3:** Suitability of the HepG2 cell line in various spheres of biomedical research.

Type of Research	Advantages	Disadvantages	Recommendations	Valid References ^1^
Toxicity tests	Including albumin and AFP^2^; No hepatitis B viral genome.	Low expression and activity levels of the drug-metabolizing enzymes [45]; There are exceptions such as $\mathrm{NQO}1^{3}$$,\;\mathrm{GSTM}3^{4}$, $\mathrm{and}\;\mathrm{MRP}1^{5}$ [127,172,173]; The minor phase I enzymes: CYP27B1, CYP2W1 are expressed at significantly higher elevated levels than in human hepatocytes [172].	According to the article (Ren et al.), the HepG2 cell line may not be a suitable model in investigating metabolism-mediated toxicity without additional modification due to it is lack of metabolic capability [174,175]. For example, the cell line can be used in studies of CYP inducers [176].	No valid experiment.
Drug metabolism				No valid experiment.
HB model	CTNNB1 exon 3 mutation [177]; Gene expression profiling demonstrated cell growth and survival pathways deregulation, similar to that of fetal and embryonal HB [44]; Losses of the chromosome 4q3 region associated with the t(1;4) translocation—translocation in HB [43].		The correct use of HepG2 is an HB model, as it has many of the characteristics of HB. According to the article (Lopez-Terrada D et al.), “the correct attribution of the tumor of origin of this cell line is of crucial interest for investigators studying the biology of hepatocellular neoplasms, particularly those engaged in novel biology-based classifications, clinical stratification, and therapeutic interventions for pediatric and adult patient” [29].	[178,179,180,181,182,183]
HCC model	Partially similar genome and transcriptome profiles.	At the genomic and transcriptomic levels has been shown to be similar to hepatoblastoma.	According to meta-analysis, the cell line has some similar mutations to HCC. However, there are a large number of cell lines derived from HCC cells, such as Huh-7, HepaRG, etc. Additionally, their use is relevant, as there are more overlaps in the genetic and transcriptome profiles.	Questionable.
Hepatocyte model	No advantages.	A large number of tumor mutations affect further levels of information transfer; The basal gene expression level, HepaRG cells are closer to primary hepatocytes compared with HepG2 cells [133].	There are non-tumor cell lines such as THLE-2 and THLE-3 that have characteristics similar to hepatocytes [22].	No valid experiment.

“Valid experiment”1 —were chosen based on the recommendations that were found in the articles; A2FP —α-fetoprotein; N3QO1 —NAD(P)H quinone dehydrogenase 1; G4STM3—glutathione S-transferase Mu 3; M5RP1—multidrug resistance protein 1.

## Supplementary Materials

All data are available online at https://www.mdpi.com/article/10.3390/ijms222313135/s1.

## References

[1] Kaur G., Dufour J.M. (2012). Cell Lines. Spermatogenesis.

[2] Wang L., Cao D., Wei C., Meng X.-J., Jiang X., Tan M. (2014). A Dual Vaccine Candidate against Norovirus and Hepatitis E Virus. Vaccine.

[3] Yang Y.-J., Pang X., Wang B., Yang J., Chen X.-J., Sun X.-G., Li Q., Zhang J., Guo B.-L., Ma B.-P. (2020). Steroidal Saponins from Trillium Tschonoskii Rhizomes and Their Cytotoxicity against HepG2 Cells. Steroids.

[4] Khanal T., Kim H.G., Hwang Y.P., Kong M.J., Kang M.J., Yeo H.K., Kim D.H., Jeong T.C., Jeong H.G. (2011). Role of Metabolism by the Human Intestinal Microflora in Arbutin-Induced Cytotoxicity in HepG2 Cell Cultures. Biochem. Biophys. Res. Commun..

[5] Steinbrecht S., König R., Schmidtke K.-U., Herzog N., Scheibner K., Krüger-Genge A., Jung F., Kammerer S., Küpper J.-H. (2019). Metabolic Activity Testing Can Underestimate Acute Drug Cytotoxicity as Revealed by HepG2 Cell Clones Overexpressing Cytochrome P450 2C19 and 3A4. Toxicology.

[6] Yokoyama Y., Sasaki Y., Terasaki N., Kawataki T., Takekawa K., Iwase Y., Shimizu T., Sanoh S., Ohta S. (2018). Comparison of Drug Metabolism and Its Related Hepatotoxic Effects in HepaRG, Cryopreserved Human Hepatocytes, and HepG2 Cell Cultures. Biol. Pharm. Bull..

[7] Chen X.-Y., Li J., Ma J., Duan F., Zhong P. (2006). Potential Role of Novel Hepatocellular Carcinoma-Associated Gene IDD01 in Promoting Tumorigenesis of HepG2 Cell Line. Chin. Med. J..

[8] Wendt H., Hillmer A., Reimers K., Kuhbier J.W., Schäfer-Nolte F., Allmeling C., Kasper C., Vogt P.M. (2011). Artificial Skin—Culturing of Different Skin Cell Lines for Generating an Artificial Skin Substitute on Cross-Weaved Spider Silk Fibres. PLoS ONE.

[9] Joseph S.C., Blackman B.A., Kelly M.L., Phillips M., Beaury M.W., Martinez I., Parronchi C.J., Bitsaktsis C., Blake A.D., Sabatino D. (2014). Synthesis, Characterization, and Biological Activity of Poly(Arginine)-Derived Cancer-Targeting Peptides in HepG2 Liver Cancer Cells. J. Pept. Sci..

[10] Frevert U., Galinski M.R., Hügel F.U., Allon N., Schreier H., Smulevitch S., Shakibaei M., Clavijo P. (1998). Malaria Circumsporozoite Protein Inhibits Protein Synthesis in Mammalian Cells. EMBO J..

[11] Liver Regeneration: Biological and Pathological Mechanisms and Implications. https://www.nature.com/articles/s41575-020-0342-4.

[12] Wiśniewski J.R., Vildhede A., Norén A., Artursson P. (2016). In-Depth Quantitative Analysis and Comparison of the Human Hepatocyte and Hepatoma Cell Line HepG2 Proteomes. J. Proteom..

[13] Trefts E., Gannon M., Wasserman D.H. (2017). The Liver. Curr. Biol..

[14] Genetic Liver Disease in Adults. https://pubmed.ncbi.nlm.nih.gov/10689414/.

[15] Castelli G., Pelosi E., Testa U. (2017). Liver Cancer: Molecular Characterization, Clonal Evolution and Cancer Stem Cells. Cancers.

[16] Sharma A., Nagalli S. (2021). Chronic Liver Disease. StatPearls.

[17] Settivari R.S., Rowlands J.C., Wilson D.M., Arnold S.M., Spencer P.J., Faqi A.S. (2017). Chapter 32—Application of Evolving Computational and Biological Platforms for Chemical Safety Assessment. A Comprehensive Guide to Toxicology in Nonclinical Drug Development.

[18] Olsavsky Goyak K.M., Laurenzana E.M., Omiecinski C.J. (2010). Hepatocyte Differentiation. Methods Mol. Biol..

[19] Tanimizu N., Mitaka T. (2016). Morphogenesis of Liver Epithelial Cells. Hepatol. Res..

[20] Nikolic M., Sustersic T., Filipovic N. (2018). In Vitro Models and On-Chip Systems: Biomaterial Interaction Studies With Tissues Generated Using Lung Epithelial and Liver Metabolic Cell Lines. Front. Bioeng. Biotechnol..

[21] Perugorria M.J., Olaizola P., Banales J.M. (2019). Cholangiocyte-to-Hepatocyte Differentiation: A Context-Dependent Process and an Opportunity for Regenerative Medicine. Hepatology.

[22] Pfeifer A.M., Cole K.E., Smoot D.T., Weston A., Groopman J.D., Shields P.G., Vignaud J.M., Juillerat M., Lipsky M.M., Trump B.F. (1993). Simian Virus 40 Large Tumor Antigen-Immortalized Normal Human Liver Epithelial Cells Express Hepatocyte Characteristics and Metabolize Chemical Carcinogens. Proc. Natl. Acad. Sci. USA.

[23] Godoy P., Hewitt N.J., Albrecht U., Andersen M.E., Ansari N., Bhattacharya S., Bode J.G., Bolleyn J., Borner C., Böttger J. (2013). Recent Advances in 2D and 3D in Vitro Systems Using Primary Hepatocytes, Alternative Hepatocyte Sources and Non-Parenchymal Liver Cells and Their Use in Investigating Mechanisms of Hepatotoxicity, Cell Signaling and ADME. Arch. Toxicol..

[24] Verrier E.R., Colpitts C.C., Schuster C., Zeisel M.B., Baumert T.F. (2016). Cell Culture Models for the Investigation of Hepatitis B and D Virus Infection. Viruses.

[25] Verrier E.R., Colpitts C.C., Bach C., Heydmann L., Weiss A., Renaud M., Durand S.C., Habersetzer F., Durantel D., Abou-Jaoudé G. (2016). A Targeted Functional RNA Interference Screen Uncovers Glypican 5 as an Entry Factor for Hepatitis B and D Viruses. Hepatology.

[26] Gripon P., Rumin S., Urban S., Le Seyec J., Glaise D., Cannie I., Guyomard C., Lucas J., Trepo C., Guguen-Guillouzo C. (2002). Infection of a Human Hepatoma Cell Line by Hepatitis B Virus. Proc. Natl. Acad. Sci. USA.

[27] Monteil M., Migianu-Griffoni E., Sainte-Catherine O., Di Benedetto M., Lecouvey M. (2014). Bisphosphonate Prodrugs: Synthesis and Biological Evaluation in HuH7 Hepatocarcinoma Cells. Eur. J. Med. Chem..

[28] Cavalloni G., Peraldo-Neia C., Varamo C., Casorzo L., Dell’Aglio C., Bernabei P., Chiorino G., Aglietta M., Leone F. (2016). Establishment and Characterization of a Human Intrahepatic Cholangiocarcinoma Cell Line Derived from an Italian Patient. Tumour. Biol..

[29] López-Terrada D., Cheung S.W., Finegold M.J., Knowles B.B. (2009). Hep G2 Is a Hepatoblastoma-Derived Cell Line. Hum. Pathol..

[30] Zhou B., Ho S.S., Greer S.U., Spies N., Bell J.M., Zhang X., Zhu X., Arthur J.G., Byeon S., Pattni R. (2019). Haplotype-Resolved and Integrated Genome Analysis of the Cancer Cell Line HepG2. Nucleic Acids Res..

[31] Saravanakumar A. (2019). An Omics Based Approach for the Identification of Biomarkers of Non-Alcoholic Fatty Liver Using in Vitro Models of Hepatic Steatosis. Ph.D Thesis.

[32] Buick J.K., Williams A., Meier M.J., Swartz C.D., Recio L., Gagné R., Ferguson S.S., Engelward B.P., Yauk C.L. (2021). A Modern Genotoxicity Testing Paradigm: Integration of the High-Throughput CometChip® and the TGx-DDI Transcriptomic Biomarker in Human HepaRG^TM^ Cell Cultures. Front. Public Health.

[33] HepaRG^TM^ Cells. https://www.thermofisher.com/order/catalog/product/HPRGC10.

[34] Yu K., Chen B., Aran D., Charalel J., Yau C., Wolf D.M., van ’t Veer L.J., Butte A.J., Goldstein T., Sirota M. (2019). Comprehensive Transcriptomic Analysis of Cell Lines as Models of Primary Tumors across 22 Tumor Types. Nat. Commun..

[35] Qiu Z., Li H., Zhang Z., Zhu Z., He S., Wang X., Wang P., Qin J., Zhuang L., Wang W. (2019). A Pharmacogenomic Landscape in Human Liver Cancers. Cancer Cell.

[36] Kasai F., Hirayama N., Ozawa M., Satoh M., Kohara A. (2018). HuH-7 Reference Genome Profile: Complex Karyotype Composed of Massive Loss of Heterozygosity. Hum. Cell.

[37] Kawamoto M., Yamaji T., Saito K., Shirasago Y., Satomura K., Endo T., Fukasawa M., Hanada K., Osada N. (2020). Identification of Characteristic Genomic Markers in Human Hepatoma HuH-7 and Huh7.5.1-8 Cell Lines. Front. Genet..

[38] Yu M., Selvaraj S.K., Liang-Chu M.M.Y., Aghajani S., Busse M., Yuan J., Lee G., Peale F., Klijn C., Bourgon R. (2015). A Resource for Cell Line Authentication, Annotation and Quality Control. Nature.

[39] Heffelfinger S.C., Hawkins H.H., Barrish J., Taylor L., Darlington G.J. (1992). SK HEP-1: A Human Cell Line of Endothelial Origin. Vitr. Cell Dev. Biol..

[40] Davies H., Bignell G.R., Cox C., Stephens P., Edkins S., Clegg S., Teague J., Woffendin H., Garnett M.J., Bottomley W. (2002). Mutations of the BRAF Gene in Human Cancer. Nature.

[41] Aden D.P., Fogel A., Plotkin S., Damjanov I., Knowles B.B. (1979). Controlled Synthesis of HBsAg in a Differentiated Human Liver Carcinoma-Derived Cell Line. Nature.

[42] Tai Y., Gao J.-H., Zhao C., Tong H., Zheng S.-P., Huang Z.-Y., Liu R., Tang C.-W., Li J. (2018). SK-Hep1: Not Hepatocellular Carcinoma Cells but a Cell Model for Liver Sinusoidal Endothelial Cells. Int. J. Clin. Exp. Pathol..

[43] Tomlinson G.E., Douglass E.C., Pollock B.H., Finegold M.J., Schneider N.R. (2005). Cytogenetic Evaluation of a Large Series of Hepatoblastomas: Numerical Abnormalities with Recurring Aberrations Involving 1q12-Q21. Genes Chromosomes Cancer.

[44] Adesina A.M., Lopez-Terrada D., Wong K.K., Gunaratne P., Nguyen Y., Pulliam J., Margolin J., Finegold M.J. (2009). Gene Expression Profiling Reveals Signatures Characterizing Histologic Subtypes of Hepatoblastoma and Global Deregulation in Cell Growth and Survival Pathways. Hum. Pathol..

[45] Guengerich F.P. (2019). Cytochrome P450 Research and The Journal of Biological Chemistry. J. Biol. Chem..

[46] Pareek A., Godavarthi A., Issarani R., Nagori B.P. (2013). Antioxidant and Hepatoprotective Activity of Fagonia Schweinfurthii (Hadidi) Hadidi Extract in Carbon Tetrachloride Induced Hepatotoxicity in HepG2 Cell Line and Rats. J. Ethnopharmacol..

[47] Ringelhan M., Pfister D., O’Connor T., Pikarsky E., Heikenwalder M. (2018). The Immunology of Hepatocellular Carcinoma. Nat. Immunol..

[48] Sung H., Ferlay J., Siegel R.L., Laversanne M., Soerjomataram I., Jemal A., Bray F. (2021). Global Cancer Statistics 2020: GLOBOCAN Estimates of Incidence and Mortality Worldwide for 36 Cancers in 185 Countries. CA Cancer J. Clin..

[49] Ananthakrishnan A., Gogineni V., Saeian K. (2006). Epidemiology of Primary and Secondary Liver Cancers. Semin. Interv. Radiol..

[50] Angelico R., Grimaldi C., Saffioti M.C., Castellano A., Spada M. (2018). Hepatocellular Carcinoma in Children: Hepatic Resection and Liver Transplantation. Transl. Gastroenterol. Hepatol..

[51] Eichenmüller M., Trippel F., Kreuder M., Beck A., Schwarzmayr T., Häberle B., Cairo S., Leuschner I., Von Schweinitz D., Strom T.M. (2014). The Genomic Landscape of Hepatoblastoma and Their Progenies with HCC-like Features. J Hepatol..

[52] Aguiar T.F.M., Rivas M.P., Costa S., Maschietto M., Rodrigues T., Sobral de Barros J., Barbosa A.C., Valieris R., Fernandes G.R., Bertola D.R. (2020). Insights Into the Somatic Mutation Burden of Hepatoblastomas From Brazilian Patients. Front. Oncol..

[53] Armengol C., Cairo S., Fabre M., Buendia M.A. (2011). Wnt Signaling and Hepatocarcinogenesis: The Hepatoblastoma Model. Int. J. Biochem. Cell Biol..

[54] Tornesello M.L., Buonaguro L., Tatangelo F., Botti G., Izzo F., Buonaguro F.M. (2013). Mutations in TP53, CTNNB1 and PIK3CA Genes in Hepatocellular Carcinoma Associated with Hepatitis B and Hepatitis C Virus Infections. Genomics.

[55] Javanmard D., Najafi M., Babaei M.R., Karbalaie Niya M.H., Esghaei M., Panahi M., Safarnezhad Tameshkel F., Tavakoli A., Jazayeri S.M., Ghaffari H. (2020). Investigation of CTNNB1 Gene Mutations and Expression in Hepatocellular Carcinoma and Cirrhosis in Association with Hepatitis B Virus Infection. Infect Agent Cancer.

[56] Tarnow G., McLachlan A. (2021). β-Catenin Signaling Regulates the In Vivo Distribution of Hepatitis B Virus Biosynthesis across the Liver Lobule. J. Virol..

[57] Huang D.-S., Wang Z., He X.-J., Diplas B.H., Yang R., Killela P.J., Liang J., Meng Q., Ye Z.-Y., Wang W. (2015). Recurrent TERT Promoter Mutations Identified in a Large-Scale Study of Multiple Tumor Types Are Associated with Increased TERT Expression and Telomerase Activation. Eur. J. Cancer.

[58] Nault J.-C., Ningarhari M., Rebouissou S., Zucman-Rossi J. (2019). The Role of Telomeres and Telomerase in Cirrhosis and Liver Cancer. Nat. Rev. Gastroenterol. Hepatol..

[59] Patel B., Taiwo R., Kim A.H., Dunn G.P. (2020). TERT, a Promoter of CNS Malignancies. Neurooncol. Adv..

[60] Nault J.C., Mallet M., Pilati C., Calderaro J., Bioulac-Sage P., Laurent C., Laurent A., Cherqui D., Balabaud C., Zucman-Rossi J. (2013). High Frequency of Telomerase Reverse-Transcriptase Promoter Somatic Mutations in Hepatocellular Carcinoma and Preneoplastic Lesions. Nat. Commun..

[61] Lombardo D., Saitta C., Giosa D., Di Tocco F.C., Musolino C., Caminiti G., Chines V., Franzè M.S., Alibrandi A., Navarra G. (2020). Frequency of Somatic Mutations in TERT Promoter, TP53 and CTNNB1 Genes in Patients with Hepatocellular Carcinoma from Southern Italy. Oncol. Lett..

[62] Muller P.A.J., Vousden K.H. (2013). P53 Mutations in Cancer. Nat. Cell Biol..

[63] Bossi G., Lapi E., Strano S., Rinaldo C., Blandino G., Sacchi A. (2006). Mutant P53 Gain of Function : Reduction of Tumor Malignancy of Human Cancer Cell Lines through Abrogation of Mutant P53 Expression. Oncogene.

[64] Villanueva A., Hoshida Y. (2011). Depicting the Role of TP53 in Hepatocellular Carcinoma Progression. J. Hepatol..

[65] Simbolo M., Vicentini C., Ruzzenente A., Brunelli M., Conci S., Fassan M., Mafficini A., Rusev B., Corbo V., Capelli P. (2018). Genetic Alterations Analysis in Prognostic Stratified Groups Identified TP53 and ARID1A as Poor Clinical Performance Markers in Intrahepatic Cholangiocarcinoma. Sci. Rep..

[66] Ruzzenente A., Fassan M., Conci S., Simbolo M., Lawlor R.T., Pedrazzani C., Capelli P., D’Onofrio M., Iacono C., Scarpa A. (2016). Cholangiocarcinoma Heterogeneity Revealed by Multigene Mutational Profiling: Clinical and Prognostic Relevance in Surgically Resected Patients. Ann. Surg. Oncol..

[67] Dakkak W. (2017). AACR Project GENIE: Powering Precision M. Physiol. Behav..

[68] Wang L., Zhu H., Zhao Y., Pan Q., Mao A., Zhu W., Zhang N., Lin Z., Zhou J., Wang Y. (2020). Comprehensive Molecular Profiling of Intrahepatic Cholangiocarcinoma in the Chinese Population and Therapeutic Experience. J. Transl. Med..

[69] Buettner S., Van Vugt J.L.A., Ijzermans J.N.M., Koerkamp B.G. (2017). Intrahepatic Cholangiocarcinoma: Current Perspectives. OncoTargets Ther..

[70] Perugorria M.J., Olaizola P., Labiano I., Esparza-Baquer A., Marzioni M., Marin J.J.G., Bujanda L., Banales J.M. (2019). Wnt-β-Catenin Signalling in Liver Development, Health and Disease. Nat. Rev. Gastroenterol. Hepatol..

[71] Wang W., Smits R., Hao H., He C. (2019). Wnt/β-Catenin Signaling in Liver Cancers. Cancers.

[72] Wang X., Wang Q. (2018). Alpha-Fetoprotein and Hepatocellular Carcinoma Immunity. Can. J. Gastroenterol. Hepatol..

[73] Chen J., Wang J., Cao D., Yang J., Shen K., Huang H., Shi X. (2021). Alpha-Fetoprotein (AFP)-Producing Epithelial Ovarian Carcinoma (EOC): A Retrospective Study of 27 Cases. Arch. Gynecol. Obs..

[74] Zhu M., Lu Y., Li W., Guo J., Dong X., Lin B., Chen Y., Xie X., Li M. (2016). Hepatitis B Virus X Protein Driven Alpha Fetoprotein Expression to Promote Malignant Behaviors of Normal Liver Cells and Hepatoma Cells. J. Cancer.

[75] Houwelingen L.V., Sandoval J.A. (2016). Alpha-Fetoprotein in Malignant Pediatric Conditions.

[76] Toyoda H., Bregerie O., Vallet A., Nalpas B., Pivert G., Brechot C., Desdouets C. (2005). Changes to Hepatocyte Ploidy and Binuclearity Profiles during Human Chronic Viral Hepatitis. Gut.

[77] (2010). The Ploidy Conveyor of Mature Hepatocytes as a Source of Genetic Variation. Nature.

[78] Li H., Feng Z., Tsang T.C., Tang T., Jia X., He X., Pennington M.E., Badowski M.S., Liu A.K.M., Chen D. (2014). Fusion of HepG2 Cells with Mesenchymal Stem Cells Increases Cancer-Associated and Malignant Properties: An in Vivo Metastasis Model. Oncol. Rep..

[79] Sun L., Hui L. (2020). Progress in Human Liver Organoids. J. Mol. Cell Biol..

[80] Weir E.G., Ali S.Z. (2002). Hepatoblastoma: Cytomorphologic Characteristics in Serous Cavity Fluids. Cancer.

[81] De Sánchez V.C., Chávez E., Velasco-Loyden G., Lozano-Rosas M.G., Aparicio-Cadena A.R. (2016). Interaction of Mitochondrial and Epigenetic Regulation in Hepatocellular Carcinoma. Liver Cancer.

[82] Wei J., Fang D. (2021). Endoplasmic Reticulum Stress Signaling and the Pathogenesis of Hepatocarcinoma. Int. J. Mol. Sci..

[83] Chen H.L., Chiu T.S., Chen P.J., Chen D.S. (1993). Cytogenetic Studies on Human Liver Cancer Cell Lines. Cancer Genet. Cytogenet..

[84] Wang X., Li T., Su X., Li J., Li W., Gan J., Wu T., Kong L., Zhang T., Tang M. (2019). Genotoxic Effects of Silver Nanoparticles with/without Coating in Human Liver HepG2 Cells and in Mice. J. Appl. Toxicol..

[85] Lee C., Ling Q., Chang M. (2014). Characterization of De Novo Genomic Alterations in HBV-Producing Characterization of De Novo Genomic Alterations in HBV-Producing Hepatoma Cells Using Array Comparative Genomic Hybridization. J. Cancer Mol..

[86] Simon D., Aden D.P., Knowles B.B. (1982). Chromosomes of Human Hepatoma Cell Lines. Int. J. Cancer.

[87] Wong N., Lai P., Pang E., Wai-Tong Leun T., Wan-Yee Lau J., James Johnson P. (2000). A Comprehensive Karyotypic Study on Human Hepatocellular Carcinoma by Spectral Karyotyping. Hepatology.

[88] Luquet I., Laı J.L. (2008). Hyperdiploid Karyotypes in Acute Myeloid Leukemia Define a Novel Entity : A Study of 38 Patients from the Groupe Francophone de Cytogenetique Hematologique (GFCH). Leukemia.

[89] Heim S., Alimena G., Billstrm R., Diverio D., Kristoffersson U., Mandahl N., Nanni M., Mitelman F. (1987). Tetraploid Karyotype (92,XXYY) in Two Patients with Acute Lymphoblastic Leukemia Sverre. Cancer Genet. Cytogenet..

[90] Wiśniewski J.R., Hein M.Y., Cox J., Mann M. (2014). A “Proteomic Ruler” for Protein Copy Number and Concentration Estimation without Spike-in Standards. Mol. Cell. Proteom..

[91] Yang T., Wen Y., Li J., Tan T., Yang J., Pan J., Hu C., Yao Y., Zhang J., Xin Y. (2019). NRAS and KRAS Polymorphisms Are Not Associated with Hepatoblastoma Susceptibility in Chinese Children. Exp. Hematol. Oncol..

[92] Yang M.H., Yen C.H., Chen Y.F., Fang C.C., Li C.H., Lee K.J., Lin Y.H., Weng C.H., Liu T.T., Huang S.F. (2019). Somatic Mutations of PREX2 Gene in Patients with Hepatocellular Carcinoma. Sci. Rep..

[93] Bévant K., Coulouarn C. (2017). Landscape of Genomic Alterations in Hepatocellular Carcinoma: Current Knowledge and Perspectives for Targeted Therapies. Hepato. Biliary Surg. Nutr..

[94] Li L., Halpert G., Lerner M.G., Hu H., Dimitrion P., Weiss M.J., He J., Philosophe B., Burkhart R., Burns W.R. (2021). Protein Synthesis Inhibitor Omacetaxine Is Effective against Hepatocellular Carcinoma. JCI Insight.

[95] Ally A., Balasundaram M., Carlsen R., Chuah E., Clarke A., Dhalla N., Holt R.A., Jones S.J.M., Lee D., Ma Y. (2017). Comprehensive and Integrative Genomic Characterization of Hepatocellular Carcinoma. Cell.

[96] Cevik D. (2015). Common Telomerase Reverse Transcriptase Promoter Mutations in Hepatocellular Carcinomas from Different Geographical Locations. World J. Gastroenterol..

[97] Sumazin P., Chen Y., Treviño L.R., Sarabia S.F., Hampton O.A., Patel K., Mistretta T.-A., Zorman B., Thompson P., Heczey A. (2017). Genomic Analysis of Hepatoblastoma Identifies Distinct Molecular and Prognostic Subgroups. Hepatology.

[98] Cagatay T., Ozturk M. (2002). P53 Mutation as a Source of Aberrant Beta-Catenin Accumulation in Cancer Cells. Oncogene.

[99] Woodfield S.E., Shi Y., Patel R.H., Chen Z., Shah A.P., Srivastava R.K., Whitlock R.S., Ibarra A.M., Larson S.R., Sarabia S.F. (2021). MDM4 Inhibition: A Novel Therapeutic Strategy to Reactivate P53 in Hepatoblastoma. Sci. Rep..

[100] Hussain S.P., Schwank J., Staib F., Wang X.W., Harris C.C. (2007). TP53 Mutations and Hepatocellular Carcinoma: Insights into the Etiology and Pathogenesis of Liver Cancer. Oncogene.

[101] Pyatnitskiy M.A., Arzumanian V.A., Radko S.P., Ptitsyn K.G., Vakhrushev I.V., Poverennaya E.V., Ponomarenko E.A. (2021). Oxford Nanopore MinION Direct RNA-Seq for Systems Biology. Biology.

[102] Tyakht A.V., Ilina E.N., Alexeev D.G., Ischenko D.S., Gorbachev A.Y., Semashko T.A., Larin A.K., Selezneva O.V., Kostryukova E.S., Karalkin P.A. (2014). RNA-Seq Gene Expression Profiling of HepG2 Cells: The Influence of Experimental Factors and Comparison with Liver Tissue. BMC Genom..

[103] Nasution A.A., Adnindya M.R., Indriyani, Liem I.K. (2017). Roles of DLK1 in Liver Development and Oncogenesis. OnLine J. Biol. Sci..

[104] Xu X., Liu R.F., Zhang X., Huang L.Y., Chen F., Fei Q.L., Han Z.G. (2012). DLK1 as a Potential Target against Cancer Stem/Progenitor Cells of Hepatocellular Carcinoma. Mol. Cancer Ther..

[105] Luo J., Ren B., Keryanov S., Tseng G.C., Rao U.N.M., Monga S.P., Strom S., Demetris A.J., Nalesnik M., Yan P. (2007). Transcriptomic and Genomic Analysis of Human Hepatocellular Carcinomas and Hepatoblastomas. Hepatology.

[106] Pivonello C., Negri M., De Martino M.C., Napolitano M., de Angelis C., Provvisiero D.P., Cuomo G., Auriemma R.S., Simeoli C., Izzo F. (2016). The Dual Targeting of Insulin and Insulin-like Growth Factor 1 Receptor Enhances the MTOR Inhibitor-Mediated Antitumor Efficacy in Hepatocellular Carcinoma. Oncotarget.

[107] Zhao H., Desai V., Wang J., Epstein D.M., Miglarese M., Buck E. (2012). Epithelial—Mesenchymal Transition Predicts Sensitivity to the Dual IGF-1R / IR Inhibitor OSI-906 in Hepatocellular Carcinoma Cell Lines. Mol. Cancer Ther..

[108] Martinez-Quetglas I., Pinyol R., Dauch D., Torrecilla S., Tovar V., Moeini A., Alsinet C., Portela A., Rodriguez-Carunchio L., Solé M. (2016). IGF2 Is Up-Regulated by Epigenetic Mechanisms in Hepatocellular Carcinomas and Is an Actionable Oncogene Product in Experimental Models. Gastroenterology.

[109] Thomas H. (2016). Liver Cancer: IGF2—An Epigenetic Oncodriver in HCC. Nat. Rev. Gastroenterol. Hepatol..

[110] Honda S., Arai Y., Haruta M., Sasaki F., Ohira M., Yamaoka H., Horie H., Nakagawara A., Hiyama E., Todo S. (2008). Loss of Imprinting of IGF2 Correlates with Hypermethylation of the H19 Differentially Methylated Region in Hepatoblastoma. Br. J. Cancer.

[111] Ekström T.J. (2000). Altered Expression of Members of the IGF-Axis in Hepatoblastoma. Br. J. Cancer.

[112] Weiss J.B.W., Wagner A.E., Eberherr C., Haberle B., Vokuhl C., Von Schweinitz D., Kappler R. (2020). High Expression of IGF2-Derived Intronic MiR-483 Predicts Outcome in Hepatoblastoma. Cancer Biomark..

[113] Shen G., Shen H., Zhang J., Yan Q., Liu H. (2020). DNA Methylation in Hepatoblastoma-a Literature Review. Ital. J. Pediatr..

[114] Nussbaum T., Samarin J., Ehemann V., Bissinger M., Ryschich E., Khamidjanov A., Yu X., Gretz N., Schirmacher P., Breuhahn K. (2008). Autocrine Insulin-like Growth Factor-II Stimulation of Tumor Cell Migration Is a Progression Step in Human Hepatocarcinogenesis. Hepatology.

[115] Waly A.A., El-Ekiaby N., Assal R.A., Abdelrahman M.M., Hosny K.A., El Tayebi H.M., Esmat G., Breuhahn K., Abdelaziz A.I. (2019). Methylation in MIRLET7A3 Gene Induces the Expression of IGF-II and Its MRNA Binding Proteins IGF2BP-2 and 3 in Hepatocellular Carcinoma. Front. Physiol..

[116] Breuhahn K., Longerich T., Schirmacher P. (2006). Dysregulation of Growth Factor Signaling in Human Hepatocellular Carcinoma. Oncogene.

[117] Chen L., Li M., Li Q., Wang C., Xie S. (2013). DKK1 Promotes Hepatocellular Carcinoma Cell Migration and Invasion through β-Catenin/MMP7 Signaling Pathway. Mol. Cancer.

[118] Wang S., Chen N., Chen Y., Sun L., Li L., Liu H. (2018). Elevated GPC3 Level Promotes Cell Proliferation in Liver Cancer. Oncol. Lett..

[119] Jia H.L., Ye Q.H., Qin L.X., Budhu A., Forgues M., Chen Y., Liu Y.K., Sun H.C., Wang L., Lu H.Z. (2007). Gene Expression Profiling Reveals Potential Biomarkers of Human Hepatocellular Carcinoma. Clin. Cancer Res..

[120] Maass T., Sfakianakis I., Staib F., Krupp M., Galle R.P., Teufel A. (2010). Microarray-Based Gene Expression Analysis of Hepatocellular Carcinoma. Curr. Genom..

[121] Sun C.K., Chua M.S., He J., So S.K. (2011). Suppression of Glypican 3 Inhibits Growth of Hepatocellular Carcinoma Cells through Up-Regulation of TGF-Β2. Neoplasia.

[122] Sekiguchi M., Seki M., Kawai T., Yoshida K., Yoshida M., Isobe T., Hoshino N. (2020). Integrated Multiomics Analysis of Hepatoblastoma Unravels Its Heterogeneity and Provides Novel Druggable Targets. NPJ Precis. Oncol..

[123] Vastrad B., Vastrad C., Kotturshetti I. (2020). Identification of Potential Core Genes in Hepatoblastoma via Bioinformatics Analysis. Genet. Genom. Med..

[124] Xu Y., Zheng Y., Li T. (2017). Modulation of IGF2BP1 by Long Non-Coding RNA HCG11 Suppresses Apoptosis of Hepatocellular Carcinoma Cells via MAPK Signaling Transduction. Int. J. Oncol..

[125] Huang X., Zhang H., Guo X., Zhu Z., Cai H., Kong X. (2018). Insulin-like Growth Factor 2 MRNA-Binding Protein 1 ( IGF2BP1 ) in Cancer. J. Hematol. Oncol..

[126] Cao J., Mu Q., Huang H. (2018). Review Article The Roles of Insulin-Like Growth Factor 2 MRNA-Binding Protein 2 in Cancer and Cancer Stem Cells. Stem Cells Int..

[127] Gerets H.H.J., Tilmant K., Gerin B., Chanteux H., Depelchin B.O., Dhalluin S., Atienzar F.A. (2012). Characterization of Primary Human Hepatocytes, HepG2 Cells, and HepaRG Cells at the MRNA Level and CYP Activity in Response to Inducers and Their Predictivity for the Detection of Human Hepatotoxins. Cell Biol. Toxicol..

[128] Berger B., Donzelli M., Maseneni S., Boess F. (2016). Comparison of Liver Cell Models Using the Basel Phenotyping Cocktail. Front. Pharmacol..

[129] Shankar K., Sciences M., Rock L. (2014). Cytochrome P450.

[130] Choi J.M., Oh S.J., Lee S.Y., Im J.H., Oh J.M., Ryu C.S., Kwak H.C., Lee J.Y., Kang K.W., Kim S.K. (2015). HepG2 Cells as an in Vitro Model for Evaluation of Cytochrome P450 Induction by Xenobiotics. Arch. Pharmacal. Res..

[131] Ji Y., Jiang X., Qi X. (2018). Downregulation of CYP2A6 and CYP2C8 in Tumor Tissues Is Linked to Worse Overall Survival and Recurrence-Free Survival from Hepatocellular Carcinoma. BioMed Res. Int..

[132] Gupta R., Schrooders Y., Hauser D., van Herwijnen M., Albrecht W., ter Braak B., Brecklinghaus T., Castell J.V., Elenschneider L., Escher S. (2021). Comparing in Vitro Human Liver Models to in Vivo Human Liver Using RNA-Seq. Arch. Toxicol..

[133] Jennen D.G.J., Magkoufopoulou C., Ketelslegers H.B., van Herwijnen M.H.M., Kleinjans J.C.S., van Delft J.H.M. (2010). Comparison of HepG2 and HepaRG by Whole-Genome Gene Expression Analysis for the Purpose of Chemical Hazard Identification. Toxicol. Sci..

[134] Wiśniewski J.R., Koepsell H., Gizak A., Rakus D. (2015). Absolute Protein Quantification Allows Differentiation of Cell-Specific Metabolic Routes and Functions. Proteomics.

[135] Zanger U.M., Turpeinen M., Klein K., Schwab M. (2008). Functional Pharmacogenetics / Genomics of Human Cytochromes P450 Involved in Drug Biotransformation. Anal. Bioanal. Chem..

[136] Vildhede A., Wiśniewski J.R., Norén A., Karlgren M., Artursson P. (2015). Comparative Proteomic Analysis of Human Liver Tissue and Isolated Hepatocytes with a Focus on Proteins Determining Drug Exposure. J. Proteome Res..

[137] Schmidt A., Braeuning A., Ruck P., Seitz G., Armeanu-Ebinger S., Fuchs J., Warmann S.W., Schwarz M. (2011). Differential Expression of Glutamine Synthetase and Cytochrome P450 Isoforms in Human Hepatoblastoma. Toxicology.

[138] Jiang F., Chen L., Yang Y.C., Wang X.M., Wang R.Y., Li L., Wen W., Chang Y.X., Chen C.Y., Tang J. (2015). CYP3A5 Functions as a Tumor Suppressor in Hepatocellular Carcinoma by Regulating MTORC2/Akt Signaling. Cancer Res..

[139] Westerink W.M.A., Schoonen W.G.E.J. (2007). Phasea II Enzyme Levels in HepG2 Cells and Cryopreserved Primary Human Hepatocytes and Their Induction in HepG2 Cells. Toxicol. In Vitro.

[140] Lu L., Zhou J., Shi J., Peng X., Qi X., Wang Y. (2015). Drug-Metabolizing Activity, Protein and Gene Expression of UDP-Glucuronosyltransferases Are Significantly Altered in Hepatocellular Carcinoma Patients. PLoS ONE.

[141] Xie C., Yan T., Chen J., Li X., Zou J., Zhu L., Lu L. (2017). LC-MS / MS Quantification of Sulfotransferases Is Better than Conventional Immunogenic Methods in Determining Human Liver SULT Activities : Implication in Precision Medicine. Sci. Rep..

[142] Huang L., Coughtrie M.W.H., Hsu H. (2005). Down-Regulation of Dehydroepiandrosterone Sulfotransferase Gene in Human Hepatocellular Carcinoma. Mol. Cell. Endocrinol..

[143] Cells H., Qu X., Zhang Y., Zhang S., Zhai J., Gao H., Tao L. (2018). Dysregulation of BSEP and MRP2 May Play an Important Role in Isoniazid-Induced Liver Injury via the SIRT1/FXR Pathway in Rats And. Biol. Pharm. Bull..

[144] Lee T.K., Hammond C.L., Ballatori N., Lee H.C., Pharmacol T.A. (2001). Intracellular Glutathione Regulates Taurocholate Transport in HepG2 Cells Intracellular Glutathione Regulates Taurocholate Transport In. Toxicol. Appl. Pharmacol..

[145] Knisely A.S., Strautnieks S.S., Meier Y., Stieger B., Byrne J.A., Portmann B.C., Moore L., Raftos J., Arnell H., Cielecka-kuszyk J. (2006). Hepatocellular Carcinoma in Ten Children Under Five Years of Age With Bile Salt Export Pump Deficiency. Hepatology.

[146] Knisely A.S., Portmann B.C., Hill D. (2006). Letter to the Editor Deficiency of BSEP in PFIC with Hepatocellular Malignancy. Natl. Libr. Med..

[147] Pérez-Pineda S.I., Baylón-Pacheco L., Espíritu-Gordillo P., Tsutsumi V., Rosales-Encina J.L. (2021). Effect of Bile Acids on the Expression of MRP3 and MRP4: An In Vitro Study in HepG2 Cell Line. Ann. Hepatol..

[148] Chen Y., Chen Z., Feng J.H., Bin Chen Y., Liao N.S., Su Y., Zou C.Y. (2018). Metabolic Profiling of Normal Hepatocyte and Hepatocellular Carcinoma Cells via 1H Nuclear Magnetic Resonance Spectroscopy. Cell Biol. Int..

[149] Ye F., Chen C., Qin J., Liu J., Zheng A.C. (2015). Genetic Profiling Reveals an Alarming Rate of Cross-Contamination among Human Cell Lines Used in China. FASEB J..

[150] Bayet-Robert M., Loiseau D., Rio P., Demidem A., Barthomeuf C., Stepien G., Morvan D. (2010). Quantitative Two-Dimensional HRMAS 1H-NMR Spectroscopy-Based Metabolite Profiling of Human Cancer Cell Lines and Response to Chemotherapy. Magn. Reson. Med..

[151] Halama A., Möller G., Adamski J. (2011). Metabolic Signatures in Apoptotic Human Cancer Cell Lines. OMICS J. Integr. Biol..

[152] Zhang Z., Sniderman A.D., Kalant D., Vu H., Monge J.C., Tao Y., Cianflone K. (1993). The Role of Amino Acids in ApoB100 Synthesis and Catabolism in Human HepG2 Cells. J. Biol. Chem..

[153] Miyazaki T., Matsuzaki Y. (2014). Taurine and Liver Diseases: A Focus on the Heterogeneous Protective Properties of Taurine. Amino Acids.

[154] Van der Veen J.N., Kennelly J.P., Wan S., Vance J.E., Vance D.E., Jacobs R.L. (2017). The Critical Role of Phosphatidylcholine and Phosphatidylethanolamine Metabolism in Health and Disease. Biochim. Biophys. Acta (BBA)—Biomembr..

[155] Harvey A.J., Badve S., Kumar G.L. (2019). Overview of Cell Signaling Pathways in Cancer. Predictive Biomarkers in Oncology: Applications in Precision Medicine.

[156] Yip H.Y.K., Papa A. (2021). Signaling Pathways in Cancer: Therapeutic Targets, Combinatorial Treatments, and New Developments. Cells.

[157] Balzarini P., Benetti A., Invernici G., Cristini S., Zicari S., Caruso A., Gatta L.B., Berenzi A., Imberti L., Zanotti C. (2012). Transforming Growth Factor-Beta1 Induces Microvascular Abnormalities through a down-Modulation of Neural Cell Adhesion Molecule in Human Hepatocellular Carcinoma. Lab. Investig..

[158] Monga S.P. (2015). β-Catenin Signaling and Roles in Liver Homeostasis, Injury, and Tumorigenesis. Gastroenterology.

[159] Tang H., Li R.-P., Liang P., Zhou Y.-L., Wang G.-W. (2015). MiR-125a Inhibits the Migration and Invasion of Liver Cancer Cells via Suppression of the PI3K/AKT/MTOR Signaling Pathway. Oncol. Lett..

[160] Wang X.H., Liu B.R., Qu B., Xing H., Gao S.L., Yin J.M., Wang X.F., Cheng Y.Q. (2011). Silencing STAT3 May Inhibit Cell Growth through Regulating Signaling Pathway, Telomerase, Cell Cycle, Apoptosis and Angiogenesis in Hepatocellular Carcinoma: Potential Uses for Gene Therapy. Neoplasma.

[161] Wang X.-H., Chen Z.-G., Xu R.-L., Lv C.-Q., Liu J., Du B. (2017). TGF-Β1 Signaling Pathway Serves a Role in HepG2 Cell Regulation by Affecting the Protein Expression of PCNA, Gankyrin, P115, XIAP and Survivin. Oncol. Lett..

[162] Sha Y.-L., Liu S., Yan W.-W., Dong B. (2019). Wnt/β-Catenin Signaling as a Useful Therapeutic Target in Hepatoblastoma. Biosci. Rep..

[163] Liu J., Li G., Liu D., Liu J. (2014). FH535 Inhibits the Proliferation of HepG2 Cells via Downregulation of the Wnt/β-Catenin Signaling Pathway. Mol. Med. Rep..

[164] Wang X., Guo S., Zhao R., Liu Y., Yang G. (2019). STAT3-Activated Long Non-Coding RNA Lung Cancer Associated Transcript 1 Drives Cell Proliferation, Migration, and Invasion in Hepatoblastoma Through Regulation of the MiR-301b/STAT3 Axis. Hum. Gene.

[165] Cui Y., Lu P., Song G., Liu Q., Zhu D., Liu X. (2016). Involvement of PI3K/Akt, ERK and P38 Signaling Pathways in Emodin-Mediated Extrinsic and Intrinsic Human Hepatoblastoma Cell Apoptosis. Food Chem. Toxicol..

[166] Ferrín G., Guerrero M., Amado V., Rodríguez-Perálvarez M., De la Mata M. (2020). Activation of MTOR Signaling Pathway in Hepatocellular Carcinoma. Int. J. Mol. Sci..

[167] Chen J., Zaidi S., Rao S., Chen J.-S., Phan L., Farci P., Su X., Shetty K., White J., Zamboni F. (2018). Analysis of Genomes and Transcriptomes of Hepatocellular Carcinomas Identifies Mutations and Gene Expression Changes in the Transforming Growth Factor-β Pathway. Gastroenterology.

[168] Immunomodulatory TGF-β Signaling in Hepatocellular Carcinoma. https://pubmed.ncbi.nlm.nih.gov/31353124/.

[169] Archakov A., Aseev A., Bykov V., Grigoriev A., Govorun V., Ivanov V., Khlunov A., Lisitsa A., Mazurenko S., Makarov A.A. (2011). Gene-Centric View on the Human Proteome Project: The Example of the Russian Roadmap for Chromosome 18. Proteomics.

[170] Orsburn B.C. (2021). Evaluation of the Sensitivity of Proteomics Methods Using the Absolute Copy Number of Proteins in a Single Cell as a Metric. Proteomes.

[171] Ponomarenko E.A., Poverennaya E.V., Ilgisonis E.V., Pyatnitskiy M.A., Kopylov A.T., Zgoda V.G., Lisitsa A.V., Archakov A.I. (2016). The Size of the Human Proteome: The Width and Depth. Int. J. Anal. Chem..

[172] Guo L., Dial S., Shi L., Branham W., Liu J., Fang J.-L., Green B., Deng H., Kaput J., Ning B. (2011). Similarities and Differences in the Expression of Drug-Metabolizing Enzymes between Human Hepatic Cell Lines and Primary Human Hepatocytes. Drug. Metab. Dispos..

[173] Ahlin G., Hilgendorf C., Karlsson J., Szigyarto C.A.-K., Uhlén M., Artursson P. (2009). Endogenous Gene and Protein Expression of Drug-Transporting Proteins in Cell Lines Routinely Used in Drug Discovery Programs. Drug. Metab. Dispos..

[174] Zhen R., Chen S., Ning B., Guo L. (2018). Use of Liver-Derived Cell Lines for the Study of Drug-Induced Liver Injury.

[175] Upgrading HepG2 Cells with Adenoviral Vectors That Encode Drug-Metabolizing Enzymes: Application for Drug Hepatotoxicity Testing. https://pubmed.ncbi.nlm.nih.gov/27671376/.

[176] Takemura A., Gong S., Sato T., Kawaguchi M., Sekine S., Kazuki Y., Horie T., Ito K. (2021). Evaluation of Parent- and Metabolite-Induced Mitochondrial Toxicities Using CYP-Introduced HepG2 Cells. J. Pharm. Sci..

[177] López-Terrada D., Gunaratne P.H., Adesina A.M., Pulliam J., Hoang D.M., Nguyen Y., Mistretta T.-A., Margolin J., Finegold M.J. (2009). Histologic Subtypes of Hepatoblastoma Are Characterized by Differential Canonical Wnt and Notch Pathway Activation in DLK+ Precursors. Hum. Pathol..

[178] Matsumoto S., Yamamichi T., Shinzawa K., Kasahara Y., Nojima S., Kodama T., Obika S., Takehara T., Morii E., Okuyama H. (2019). GREB1 Induced by Wnt Signaling Promotes Development of Hepatoblastoma by Suppressing TGFβ Signaling. Nat. Commun..

[179] Regel I., Eichenmüller M., Mahajan U.M., Hagl B., Benitz S., Häberle B., Vokuhl C., von Schweinitz D., Kappler R. (2020). Downregulation of SFRP1 Is a Protumorigenic Event in Hepatoblastoma and Correlates with Beta-Catenin Mutations. J. Cancer Res. Clin. Oncol..

[180] Epidermal Growth Factor Receptor/Heme Oxygenase-1 Axis Is Involved in Chemoresistance to Cisplatin and Pirarubicin in HepG2 Cell Lines and Hepatoblastoma Specimens. https://pubmed.ncbi.nlm.nih.gov/31559456/.

[181] Romanelli R.G., Petrai I., Robino G., Efsen E., Novo E., Bonacchi A., Pagliai G., Grossi A., Parola M., Navari N. (2006). Thrombopoietin Stimulates Migration and Activates Multiple Signaling Pathways in Hepatoblastoma Cells. Am. J. Physiol. Gastrointest. Liver Physiol..

[182] Ye M., He J., Zhang J., Liu B., Liu X., Xie L., Wei M., Dong R., Li K., Ma D. (2021). USP7 Promotes Hepatoblastoma Progression through Activation of PI3K/AKT Signaling Pathway. Cancer Biomark..

[183] Liu G., Liu B., Liu X., Xie L., He J., Zhang J., Dong R., Ma D., Dong K., Ye M. (2021). ARID1B/SUB1-Activated LncRNA HOXA-AS2 Drives the Malignant Behaviour of Hepatoblastoma through Regulation of HOXA3. J. Cell Mol. Med..

[184] Rauch C., Jennings P., Wilmes A. (2014). Vitro Toxicology Systems.

[185] Perea L., Coll M., Sancho-Bru P. (2015). Assessment of Liver Fibrotic Insults In Vitro.

[186] Bouma M.E., Rogier E., Verthier N., Labarre C., Feldmann G. (1989). Further Cellular Investigation of the Human Hepatoblastoma-Derived Cell Line HepG2: Morphology and Immunocytochemical Studies of Hepatic-Secreted Proteins. Vitr. Cell. Dev. Biol. J. Tissue Cult. Assoc..

[187] Krithika R., Mohankumar R., Verma R.J., Shrivastav P.S., Mohamad I.L., Gunasekaran P., Narasimhan S. (2009). Isolation, Characterization and Antioxidative Effect of Phyllanthin against CCl4-Induced Toxicity in HepG2 Cell Line. Chem. Biol. Interact..

[188] Xuan J., Chen S., Ning B., Tolleson W.H., Guo L. (2017). Development of HepG2-Derived Cells Expressing Cytochrome P450s for Assessing Metabolism-Associated Drug-Induced Liver Toxicity. Physiol. Behav..

[189] Ooka M., Lynch C., Xia M. (2020). Application of in Vitro Metabolism Activation in High-Throughput Screening. Int. J. Mol. Sci..

[190] Anton D., Burckel H., Josset E., Noel G. (2015). Three-Dimensional Cell Culture: A Breakthrough in Vivo. Int. J. Mol. Sci..

[191] Shah U.K., de Mallia J.O., Singh N., Chapman K.E., Doak S.H., Jenkins G.J.S. (2018). A Three-Dimensional in Vitro HepG2 Cells Liver Spheroid Model for Genotoxicity Studies. Mutat. Res. Genet. Toxicol. Environ. Mutagenesis.

