# Supplementary material for: The Curious Case of the HepG2 Cell Line: 40 Years of Expertise
Source: Int J Mol Sci. 2021 Dec 4;22(23):13135. doi: 10.3390/ijms222313135 (PMC8658661; doi:10.3390/ijms222313135)
Supplement: Supplementary file 1 [file ijms-22-13135-s001.zip › ijms-1464996-supplementary.pdf]

Supplementary Material Table S1 Characterization of human hepatic cell lines

Cell line	Cell type	Morphology[1]	Mutated genes[2]	Number of chromosomes	Number of articles (PubMed)	References
SK-Hep-1	Adenocarcinoma	Epithelial	CDKN2A, BRAF	56-64	602	[1,2]
Huh6	Hepatoblastoma	Epithelial	CTNNB1, TP53	NA	212	[3–5]
HCC.1.1	Hepatocellular Carcinoma	Epithelial	NA	Gains: 7, 8, 12q, and 20p and losses: 4, 6, 13q and 8, 9p	2	[6,7]
HCC.2					2	
HCC.1.2					2	
HCC.3					1	[6]
Hep3B	Hepatocellular Carcinoma	Epithelial	AXIN1, RB1	≈60	2,994	[3,8]
HLE	Hepatocellular Carcinoma	Epithelial	TERT, TP53	NA	369	[14,15]
Huh7	Hepatocellular Carcinoma	Epithelial	KDR, POLD3, TERT, TP53	55-63	6,463	[3,9–11]
MHCC97H	Hepatocellular Carcinoma	Epithelial	BRD7, KEAP1, TP53	59-65	157	[9,12]

PLC.PRF5	Hepatocellular Carcinoma	Epithelial	CDKN2A, TP53, TSC2	50-60	33	[9,13,14]
SNU182	Hepatocellular Carcinoma	Epithelial	TP53	50-56	41	[15–17]
SNU354			ARID2, AXIN1, TERT	NA	18	[9,18]
SNU368			ARID1A, AXIN1, TERT, TP53	78-84	13	[9,15,19]
SNU387			NRAS, TERT, TP53	≈67	57	[19–21]
SNU398			CTNNB1, TP53	57-62	80	[9,15,19]
SNU423			TERT, TP53	≈79	33	[9,19,20,22]
SNU475				58-66	45	[15,19,20]
SNU739			ARID1B, TERT, TP53	NA	5	[9,19]
SNU761			TP53	NA	13	[9]
SNU878			TSC2, TP53	NA	2	[9,19]
SNU886			TERT, TP53	NA	3	

MT-CHC01	Intrahepatic cholangiocarcinoma	Epithelial	KRAS	52-77	4	[12,23]
THLE-2	Normal	Epithelial	No mutations	near diploid	74	[24]
THLE-3					39	
HepaRG	Hepatocellular Carcinoma	Epithelial-	PLIN2, ANXA1, H2AFY, SNX1, GCHFR, APO	46	880	[25–27]
TFK-1	Cholangiocarcinoma	Epithelial cells growing as monolayers	TP53	≈72 (64-81)	102	[28]
SNU449	Hepatocellular Carcinoma	Epithelial, diffusely spreading cells	ARID1A, AXIN1, TP53	57-63	139	[9,15,19,29]
HepG2	Hepatoblastoma	Epithelial	CTNNB1	50-60	34,021	[30,31]
JHH1	Hepatocellular Carcinoma	Epithelial	CREBBP	NA	13	[32,33]
JHH2			CDKN2A, TERT, TP53	NA	8	[9,33]
JHH4			ACVR2A, TERT, TP53	74	16	[5,34]

JHH5			AXIN1, TP53, TERT	NA	5	[9,20]
JHH6			TP53, TERT	NA	25	
JHH7				NA	23	
Li7	Hepatocellular Carcinoma	Epithelial	TERT	NA	26	[9,35]
Mahlavu	Hepatocellular Carcinoma	Fibroblastoid	PIK3CA, TP53	NA	100	[36,37]
Fa2N-4	Normal,	Hepatocytes	NA	NA	21	[38]
B1	Hepatocellular Carcinoma	NA	TP53,	NA	1	[39]
HLF	Hepatocellular Carcinoma	NA	TERT, TP53	NA	323	[23,40]
Huh1	Hepatocellular Carcinoma	NA	AXIN1, TP53	NA	57	[3,41]
LX-2	Normal	Stellate	HSC/MFB, FN1, collagen type IV, collagen type I, various TGF-β receptors, vimentin, desmin, α-smooth	55-78	801	[42,43]

			muscle actin, CTGF, and p21			
LSEC line	Normal	Sinusoidal	NA	NA	2	[44]

1-information according to ATCC (https://www.atcc.org/) and Expasy Cellosaurus (https://web.expasy.org/cellosaurus/) ; 2 – information according to Expasy Cellosaurus (https://web.expasy.org/cellosaurus/), NA – no find data in ATCC (https://www.atcc.org/) and Expasy Cellosaurus (https://web.expasy.org/cellosaurus/), PubMed (https://pubmed.ncbi.nlm.nih.gov/) – accessed on 29 November 2021.

Reference:

1. Heffelfinger, S.C.; Hawkins, H.H.; Barrish, J.; Taylor, L.; Darlington, G.J. SK HEP-1: A Human Cell Line of Endothelial Origin. *In Vitro Cell Dev Biol* **1992**, *28A*, 136–142, doi:10.1007/BF02631017.
2. Davies, H.; Bignell, G.R.; Cox, C.; Stephens, P.; Edkins, S.; Clegg, S.; Teague, J.; Woffendin, H.; Garnett, M.J.; Bottomley, W.; et al. Mutations of the BRAF Gene in Human Cancer. *Nature* **2002**, *417*, 949–954, doi:10.1038/nature00766.
3. Yu, K.; Chen, B.; Aran, D.; Charalel, J.; Yau, C.; Wolf, D.M.; van 't Veer, L.J.; Butte, A.J.; Goldstein, T.; Sirota, M. Comprehensive Transcriptomic Analysis of Cell Lines as Models of Primary Tumors across 22 Tumor Types. *Nat Commun* **2019**, *10*, 3574, doi:10.1038/s41467-019-11415-2.
4. de La Coste, A.; Romagnolo, B.; Billuart, P.; Renard, C.A.; Buendia, M.A.; Soubrane, O.; Fabre, M.; Chelly, J.; Beldjord, C.; Kahn, A.; et al. Somatic Mutations of the Beta-Catenin Gene Are Frequent in Mouse and Human Hepatocellular Carcinomas. *Proc Natl Acad Sci U S A* **1998**, *95*, 8847–8851, doi:10.1073/pnas.95.15.8847.
5. Waldherr, M.; Mišík, M.; Ferk, F.; Tomc, J.; Žegura, B.; Filipič, M.; Mikulits, W.; Mai, S.; Haas, O.; Huber, W.W.; et al. Use of HuH6 and Other Human-Derived Hepatoma Lines for the Detection of Genotoxins: A New Hope for Laboratory Animals? *Arch Toxicol* **2018**, *92*, 921–934, doi:10.1007/s00204-017-2109-4.
6. Sagmeister, S.; Eisenbauer, M.; Pirker, C.; Mohr, T.; Holzmann, K.; Zwickl, H.; Bichler, C.; Kandioler, D.; Wrba, F.; Mikulits, W.; et al. New Cellular Tools Reveal Complex Epithelial–Mesenchymal Interactions in Hepatocarcinogenesis. *Br J Cancer* **2008**, *99*, 151–159, doi:10.1038/sj.bjc.6604440.
7. A Human Model of Epithelial to Mesenchymal Transition to Monitor Drug Efficacy in Hepatocellular Carcinoma Progression | Molecular Cancer Therapeutics Available online: https://mct.aacrjournals.org/content/10/5/850.long (accessed on 25 November 2021).
8. Yu, M.; Selvaraj, S.K.; Liang-Chu, M.M.Y.; Aghajani, S.; Busse, M.; Yuan, J.; Lee, G.; Peale, F.; Klijn, C.; Bourgon, R.; et al. A Resource for Cell Line Authentication, Annotation and Quality Control. *Nature* **2015**, *520*, 307–311, doi:10.1038/nature14397.
9. Qiu, Z.; Li, H.; Zhang, Z.; Zhu, Z.; He, S.; Wang, X.; Wang, P.; Qin, J.; Zhuang, L.; Wang, W.; et al. A Pharmacogenomic Landscape in Human Liver Cancers. *Cancer Cell* **2019**, *36*, 179-193.e11, doi:10.1016/j.ccell.2019.07.001.
10. Kasai, F.; Hirayama, N.; Ozawa, M.; Satoh, M.; Kohara, A. HuH-7 Reference Genome Profile: Complex Karyotype Composed of Massive Loss of Heterozygosity. *Hum Cell* **2018**, *31*, 261–267, doi:10.1007/s13577-018-0212-3.

11. Kawamoto, M.; Yamaji, T.; Saito, K.; Shirasago, Y.; Satomura, K.; Endo, T.; Fukasawa, M.; Hanada, K.; Osada, N. Identification of Characteristic Genomic Markers in Human Hepatoma HuH-7 and Huh7.5.1-8 Cell Lines. *Front Genet* **2020**, *11*, 546106, doi:10.3389/fgene.2020.546106.
12. Tian, J.; Tang, Z.Y.; Ye, S.L.; Liu, Y.K.; Lin, Z.Y.; Chen, J.; Xue, Q. New Human Hepatocellular Carcinoma (HCC) Cell Line with Highly Metastatic Potential (MHCC97) and Its Expressions of the Factors Associated with Metastasis. *Br J Cancer* **1999**, *81*, 814–821, doi:10.1038/sj.bjc.6690769.
13. Puisieux, A.; Galvin, K.; Troalen, F.; Bressac, B.; Marcais, C.; Galun, E.; Ponchel, F.; Yakicier, C.; Ji, J.; Ozturk, M. Retinoblastoma and P53 Tumor Suppressor Genes in Human Hepatoma Cell Lines. *FASEB J* **1993**, *7*, 1407–1413, doi:10.1096/fasebj.7.14.8224613.
14. Pinto, M.R.; Bey, E.; Bernstein, R. The PLC/PRF/5 Human Hepatoma Cell Line. I. Reevaluation of the Karyotype. *Cancer Genetics and Cytogenetics* **1985**, *18*, 11–18, doi:10.1016/0165-4608(85)90033-0.
15. Hwang, H.-J.; Kim, G.-J.; Lee, G.-B.; Oh, J.-T.; Chun, Y.-H.; Park, S.-H. A Comprehensive Karyotypic Analysis on Korean Hepatocellular Carcinoma Cell Lines by Cross-Species Color Banding and Comparative Genomic Hybridization. *Cancer Genetics and Cytogenetics* **2003**, *141*, 128–137, doi:10.1016/S0165-4608(02)00671-4.
16. Hussain, S.P.; Schwank, J.; Staib, F.; Wang, X.W.; Harris, C.C. TP53 Mutations and Hepatocellular Carcinoma: Insights into the Etiology and Pathogenesis of Liver Cancer. *Oncogene* **2007**, *26*, 2166–2176, doi:10.1038/sj.onc.1210279.
17. Park, J.G.; Lee, J.H.; Kang, M.S.; Park, K.J.; Jeon, Y.M.; Lee, H.J.; Kwon, H.S.; Park, H.S.; Yeo, K.S.; Lee, K.U. Characterization of Cell Lines Established from Human Hepatocellular Carcinoma. *Int J Cancer* **1995**, *62*, 276–282, doi:10.1002/ijc.2910620308.
18. Ku, J.-L.; Park, J.-G. Biology of SNU Cell Lines. *Cancer Res Treat* **2005**, *37*, 1–19, doi:10.4143/crt.2005.37.1.1.
19. Kang, M.S.; Lee, H.J.; Lee, J.H.; Ku, J.L.; Lee, K.P.; Kelley, M.J.; Won, Y.J.; Kim, S.T.; Park, J.G. Mutation of P53 Gene in Hepatocellular Carcinoma Cell Lines with HBX DNA. *Int J Cancer* **1996**, *67*, 898–902, doi:10.1002/(SICI)1097-0215(19960917)67:6<898::AID-IJC22>3.0.CO;2-X.
20. Ghandi, M.; Huang, F.W.; Jané-Valbuena, J.; Kryukov, G.V.; Lo, C.C.; McDonald, E.R.; Barretina, J.; Gelfand, E.T.; Bielski, C.M.; Li, H.; et al. Next-Generation Characterization of the Cancer Cell Line Encyclopedia. *Nature* **2019**, *569*, 503–508, doi:10.1038/s41586-019-1186-3.
21. SNU-387 | ATCC Available online: https://www.atcc.org/products/crl-2237 (accessed on 28 November 2021).
22. SNU-423 | ATCC Available online: https://www.atcc.org/products/crl-2238 (accessed on 28 November 2021).
23. Dor, I.; Namba, M.; Sato, J. Establishment and Some Biological Characteristics of Human Hepatoma Cell Lines. *Gan* **1975**, *66*, 385–392.
24. Pfeifer, A.M.; Cole, K.E.; Smoot, D.T.; Weston, A.; Groopman, J.D.; Shields, P.G.; Vignaud, J.M.; Juillerat, M.; Lipsky, M.M.; Trump, B.F. Simian Virus 40 Large Tumor Antigen-Immortalized Normal Human Liver Epithelial Cells Express Hepatocyte Characteristics and Metabolize Chemical Carcinogens. *Proc Natl Acad Sci U S A* **1993**, *90*, 5123–5127, doi:10.1073/pnas.90.11.5123.
25. Saravanakumar, A. An Omics Based Approach for the Identification of Biomarkers of Non-Alcoholic Fatty Liver Using in Vitro Models of Hepatic Steatosis. *Dissertations and Master's Theses (Campus Access)* **2019**, 1–184.
26. Buick, J.K.; Williams, A.; Meier, M.J.; Swartz, C.D.; Recio, L.; Gagné, R.; Ferguson, S.S.; Engelward, B.P.; Yauk, C.L. A Modern Genotoxicity Testing Paradigm: Integration of the High-Throughput CometChip® and the TGx-DDI Transcriptomic Biomarker in Human HepaRG™ Cell Cultures. *Frontiers in Public Health* **2021**, *9*, 1144, doi:10.3389/fpubh.2021.694834.
27. HepaRG™ Cells, Cryopreserved Available online: https://www.thermofisher.com/order/catalog/product/HPRGC10 (accessed on 28 November 2021).
28. Saijyo, S.; Kudo, T.; Suzuki, M.; Katayose, Y.; Shinoda, M.; Muto, T.; Fukuhara, K.; Suzuki, T.; Matsuno, S. Establishment of a New Extrahepatic Bile Duct Carcinoma Cell Line, TFK-1. *Tohoku J Exp Med* **1995**, *177*, 61–71, doi:10.1620/tjem.177.61.
29. SNU-449 | ATCC Available online: https://www.atcc.org/products/crl-2234 (accessed on 28 November 2021).
30. Wiśniewski, J.R.; Vildhede, A.; Norén, A.; Artursson, P. In-Depth Quantitative Analysis and Comparison of the Human Hepatocyte and Hepatoma Cell Line HepG2 Proteomes. *J Proteomics* **2016**, *136*, 234–247, doi:10.1016/j.jprot.2016.01.016.
31. Haplotype-Resolved and Integrated Genome Analysis of the Cancer Cell Line HepG2 Available online: https://www.ncbi.nlm.nih.gov/pmc/articles/PMC6486628/ (accessed on 28 November 2021).

32. JHH1 DepMap Cell Line Summary Available online: https://depmap.org/portal/cell_line/ACH-000620?tab=mutation (accessed on 28 November 2021).
33. Fujise, K.; Nagamori, S.; Hasumura, S.; Homma, S.; Sujino, H.; Matsuura, T.; Shimizu, K.; Niiya, M.; Kameda, H.; Fujita, K. Integration of Hepatitis B Virus DNA into Cells of Six Established Human Hepatocellular Carcinoma Cell Lines. *Hepatogastroenterology* **1990**, *37*, 457–460.
34. Nagamori, S.; Hasumura, S.; Kawada, M.; Matsuura, T.; Mizutani, S.; Yoshida, H. Human Hepatoma-Derived Cell Line FLC-4 and Method for Producing Useful Polymers by Culturing the Cell Line 1998.
35. CELL SEARCH SYSTEM -CELL BANK- (RIKEN BRC) [RCB1941 : Li-7] Available online: https://cellbank.brc.riken.jp/cell_bank/CellInfo/?cellNo=RCB1941 (accessed on 28 November 2021).
36. Oefinger, P.E.; Bronson, D.L.; Dreesman, G.R. Induction of Hepatitis B Surface Antigen in Human Hepatoma-Derived Cell Lines. *J Gen Virol* **1981**, *53*, 105–113, doi:10.1099/0022-1317-53-1-105.
37. Image Classification of Human Carcinoma Cells Using Complex Wavelet-Based Covariance Descriptors Available online: https://www.ncbi.nlm.nih.gov/pmc/articles/PMC3547046/#pone.0052807-Oefinger1 (accessed on 28 November 2021).
38. Hariparsad, N.; Carr, B.A.; Evers, R.; Chu, X. Comparison of Immortalized Fa2N-4 Cells and Human Hepatocytes as in Vitro Models for Cytochrome P450 Induction. *Drug Metab Dispos* **2008**, *36*, 1046–1055, doi:10.1124/dmd.108.020677.
39. Caruso, S.; Calatayud, A.-L.; Pilet, J.; La Bella, T.; Rekik, S.; Imbeaud, S.; Letouzé, E.; Meunier, L.; Bayard, Q.; Rohr-Udilova, N.; et al. Analysis of Liver Cancer Cell Lines Identifies Agents With Likely Efficacy Against Hepatocellular Carcinoma and Markers of Response. *Gastroenterology* **2019**, *157*, 760–776, doi:10.1053/j.gastro.2019.05.001.
40. Hsu, I.C.; Tokiwa, T.; Bennett, W.; Metcalf, R.A.; Welsh, J.A.; Sun, T.; Harris, C.C. P53 Gene Mutation and Integrated Hepatitis B Viral DNA Sequences in Human Liver Cancer Cell Lines. *Carcinogenesis* **1993**, *14*, 987–992, doi:10.1093/carcin/14.5.987.
41. Liver Cancer Cell Lines Database Available online: https://lccl.zucmanlab.com/hcc/cellLines (accessed on 25 November 2021).
42. Xu, L.; Hui, A.Y.; Albanis, E.; Arthur, M.J.; O'Byrne, S.M.; Blaner, W.S.; Mukherjee, P.; Friedman, S.L.; Eng, F.J. Human Hepatic Stellate Cell Lines, LX-1 and LX-2: New Tools for Analysis of Hepatic Fibrosis. *Gut* **2005**, *54*, 142–151, doi:10.1136/gut.2004.042127.
43. Weiskirchen, R.; Weimer, J.; Meurer, S.K.; Kron, A.; Seipel, B.; Vater, I.; Arnold, N.; Siebert, R.; Xu, L.; Friedman, S.L.; et al. Genetic Characteristics of the Human Hepatic Stellate Cell Line LX-2. *PLOS ONE* **2013**, *8*, e75692, doi:10.1371/journal.pone.0075692.
44. [Morphologic and Functional Characteristics of the Immortalized Human Liver Sinusoidal Endothelial Cell Line] - PubMed Available online: https://pubmed.ncbi.nlm.nih.gov/20302755/ (accessed on 2 December 2021).
